# Experience-Dependent Plasticity of Periglomerular Cells in the Olfactory Bulb

**DOI:** 10.1523/ENEURO.0171-26.2026

**Published:** 2026-07-14

**Authors:** Akiko Ide, Yusuke Suzuki, Itaru Imayoshi, Masayuki Sakamoto

**Affiliations:** ^1^Department of Brain Development and Regeneration, Graduate School of Biostudies, Kyoto University, Kyoto 606-8501, Japan; ^2^Center for Living Systems Information Science, Graduate School of Biostudies, Kyoto University, Kyoto 606-8501, Japan; ^3^Laboratory of Deconstruction of Stem Cells, Institute for Frontier Life and Medical Sciences, Kyoto University, Kyoto 606-8501, Japan

**Keywords:** calcium imaging, inhibitory neuron, olfactory bulb, periglomerular cells, plasticity, two-photon microscopy

## Abstract

The olfactory bulb (OB) serves as the first relay station that processes odor information. Within the OB, odor representations are dynamically modulated by reciprocal synapses between excitatory mitral and tufted (M/T) cells and local inhibitory interneurons. Among these interneurons, periglomerular cells (PGCs) are GABAergic neurons located in the glomerular layer that provide inhibitory inputs to M/T cells. Although their anatomical and molecular properties have been extensively characterized, the in vivo dynamics of PGCs during odor processing and learning remain poorly understood. To address this question, we performed in vivo two-photon calcium imaging of awake male mice to monitor the activity of individual PGCs during odor-associated tasks. PGCs exhibited robust and odor-specific responses that varied among individual odorants. Repeated passive odor exposure reduced the number of odor-responsive PGCs while preserving their odor selectivity. In contrast, active odor discrimination learning expanded the population of odor-responsive PGCs and enhanced response amplitude. Furthermore, discriminating highly similar odor mixtures recruited a broader ensemble of PGCs with lower selectivity, but this representation sharpened over training, resulting in fewer yet more selective PGCs. These findings demonstrate the experience-dependent features of PGCs and reveal their critical role in tuning olfactory processing. Our study provides fundamental insights into how inhibitory circuits regulate sensory representations underlying adaptive odor-guided behaviors.

## Significance Statement

Periglomerular cells (PGCs) in the olfactory bulb (OB) are inhibitory interneurons that modulate odor representations and regulate output from the OB to higher brain regions. Using chronic two-photon calcium imaging, we found that repeated passive odor exposure gradually reduced PGC responses, whereas the active Go/No-go odor discrimination task enhanced odor responses specifically to reward-associated odors. Moreover, when odor pairs became more similar, the population of PGCs showing excitatory or inhibitory responses to Go odors expanded across days. These results indicate that PGCs exhibit learning-dependent plasticity, enhancing responses to reward-predictive odors and thereby contributing to improved odor discrimination and adaptive behavior for future rewards.

## Introduction

Olfaction plays an essential role in adaptive behaviors, and understanding how olfactory information is processed is crucial for revealing the neural mechanisms underlying sensory perception and decision-making.

The olfactory bulb (OB) is the first central relay station that processes odor information in the brain (Shepherd, 2004). Odor molecules are detected by odorant receptors expressed on olfactory sensory neurons (OSNs) in the olfactory epithelium (Buck and Axel, 1991). OSN axons converge into glomeruli in the OB, where they form synapses onto the apical dendrites of mitral and tufted (M/T) cells, the principal output excitatory neurons in the OB (Vassar et al., 1994; Potter et al., 2001; Sakano, 2010). M/T cells project odor information to higher brain regions (Miyamichi et al., 2011; Sosulski et al., 2011; Igarashi et al., 2012; Chen et al., 2022). Previous studies have demonstrated that odor representations in M/T cells are highly dynamic and exhibit experience-dependent plasticity (Doucette and Restrepo, 2008; Kato et al., 2012; Chu et al., 2016; Yamada et al., 2017).

Inhibitory interneurons in the OB play critical roles in transforming and refining odor representations (Shepherd, 1972; Laurent, 1997; Wilson and Mainen, 2006; Kato et al., 2013; Banerjee et al., 2015; Burton, 2017; Zavitz et al., 2020). Granule cells (GCs) form dendrodendritic reciprocal synapses with the M/T cells and send GABAergic inhibitory feedback that enhances the signal-to-noise ratio of odor responses (Arevian et al., 2008; Gire and Schoppa, 2009; Najac et al., 2015; Li et al., 2018).

Periglomerular cells (PGCs) are another type of interneuron located in the glomerular layer of the OB. PGCs are predominantly GABAergic inhibitory neurons that receive inputs from OSNs and provide inhibition onto the apical dendrites of M/T cells (Aungst et al., 2003; Murphy et al., 2005; Gire and Schoppa, 2009). PGCs are structurally and physiologically heterogeneous (Kosaka and Kosaka, 2005) and can be subdivided based on expression of markers that potentially serve specific functional roles (Kosaka and Kosaka, 2007; Parrish-Aungst et al., 2007; Kiyokage et al., 2010; Nagayama et al., 2014; Capsoni et al., 2021). In addition, PGCs receive top–down inputs from the olfactory cortex including the anterior olfactory nucleus and the piriform cortex (Boyd et al., 2012; Wen et al., 2019; Mazo et al., 2022). These centrifugal inputs have been suggested to play an important role in modulating synaptic plasticity of glomerular circuits. Furthermore, the survival and functional integration of adult-born PGCs are influenced by sensory experience (Imayoshi et al., 2008; Sawada et al., 2011; Livneh et al., 2014; Sakamoto et al., 2014). This cellular diversity and experience-dependent plasticity are considered to contribute to both the sharpening and flexibility of sensory representations. Previous studies utilizing in vivo calcium imaging revealed the basic odor-evoked properties of juxtaglomerular neurons, including PGCs (Homma et al., 2013; Adam et al., 2014). However, despite extensive anatomical and molecular characterization, it remains unclear how PGC activity dynamically changes during odor processing and contributes to the experience-dependent changes in odor representations.

In this study, we performed in vivo two-photon calcium imaging of awake mice to monitor PGC activity with single-cell resolution during odor-associated tasks. We found that PGCs exhibited robust and distinct odor-evoked responses to individual odors. Then, to explore how PGC activity is shaped by odor experiences, we designed three distinct experiments. First, during a passive odor exposure paradigm without associative learning, repeated odor delivery reduced the number of odor-responsive PGCs while preserving odor selectivity. Second, during an active odor discrimination task, the number of PGCs responding to the reward-associated odor and their response amplitudes increased with learning, indicating that a larger PGC ensemble is recruited. Third, when discriminating highly similar odor mixtures, odor selectivity was initially low but improved as learning progressed. The population required for encoding odor identity became smaller after learning, indicating an experience-dependent sparsening of PGC representations. These findings reveal dynamic features of PGC plasticity and provide insights into how inhibitory circuits shape odor representations in the OB.

## Materials and Methods

### Animals

All experimental procedures were performed using protocols that were approved by the Institutional Animal Care and Use Committee at Kyoto University (protocol number, Lif-K25012). Wild-type C57BL/6N mice (Japan SLC) were group-housed and kept on a 12 h light/dark cycle with *ad libitum* food and water, and all experiments were performed in the dark period. Male mice between 4–16 weeks of age were used for all experiments, and each mouse participated in only one behavioral task.

### AAV production and injection

Recombinant adeno-associated virus (AAV) was produced using HEK293T cells (ATCC, CRL-11268) and purified by AVB Sepharose (Cytiva) as previously described (Kawashima et al., 2013). For virus injection, 4- to 5-week-old mice were anesthetized with an anesthetic mixture containing 0.075 mg/ml medetomidine hydrochloride (Zenoaq), 0.400 mg/ml midazolam (Sandoz), and 0.500 mg/ml butorphanol tartrate (Meiji Seika). This mixture was administered at 0.1 ml per 10 g of body weight (BW) intraperitoneally. For imaging PGC activity, C57BL/6N mice were injected unilaterally in the glomerular layer of the OB (AP +4.3 mm, ML −0.9 mm from the bregma; DV −0.15 mm from the pial surface) with AAV2/1-mDlx-GCaMP6f (volume, 450–600 nl; titer, 1.0 × 10^13^ genome copies/ml) at a constant rate of 30 nl/min. After the injection, carprofen (5.0 mg/kg-BW, Zoetis) was administered intraperitoneally. Mice were subjected to experiments 4 weeks after the injection.

### Cranial window implantation

Cranial window implantation was performed as described previously with minor modifications (Sakamoto et al., 2022). Mice were anesthetized with the anesthetic mixture described above. Before surgery, dexamethasone sodium phosphate (2.0 mg/kg-BW, FUJIFILM Wako Pure Chemical) and carprofen (5.0 mg/kg-BW, Zoetis) were administered to prevent inflammation and pain. During surgery, mice were put on a heating pad, and body temperature was kept at 37°C. A custom-made stainless head plate was fixed to the skull using cyanoacrylate adhesive and dental cement (Sun Medical) above the left OB. A craniotomy was drilled with a 1.0 mm diameter, and the brain was kept moist with saline. A cover glass (1.5 mm diameter, #0 thickness, Matsunami Glass) was placed over the craniotomy site with surgical adhesive glue (Aron Alpha A, Sankyo). The mice were subjected to imaging >1 week after the surgery.

### In vivo two-photon calcium imaging

In vivo two-photon calcium imaging was performed with FVMPE-RS (Olympus) equipped with a water-immersion 25× objective lens (N.A.: 1.05, Olympus) and a tunable femtosecond laser (Insight DS + Dual, Spectra-Physics) and a GaAsP photomultiplier detector (Hamamatsu Photonics). Images (339.41 × 339.41 µm^2^, 512 × 512 pixels) were acquired at 15 Hz. The laser wavelength was tuned to 940 nm, and the laser power was adjusted to 45–73 mW at the front aperture of the objective. GCaMP6f fluorescence signal was collected through a 495–540 nm bandpass filter (Olympus).

### Odor delivery

**Figure 1.**
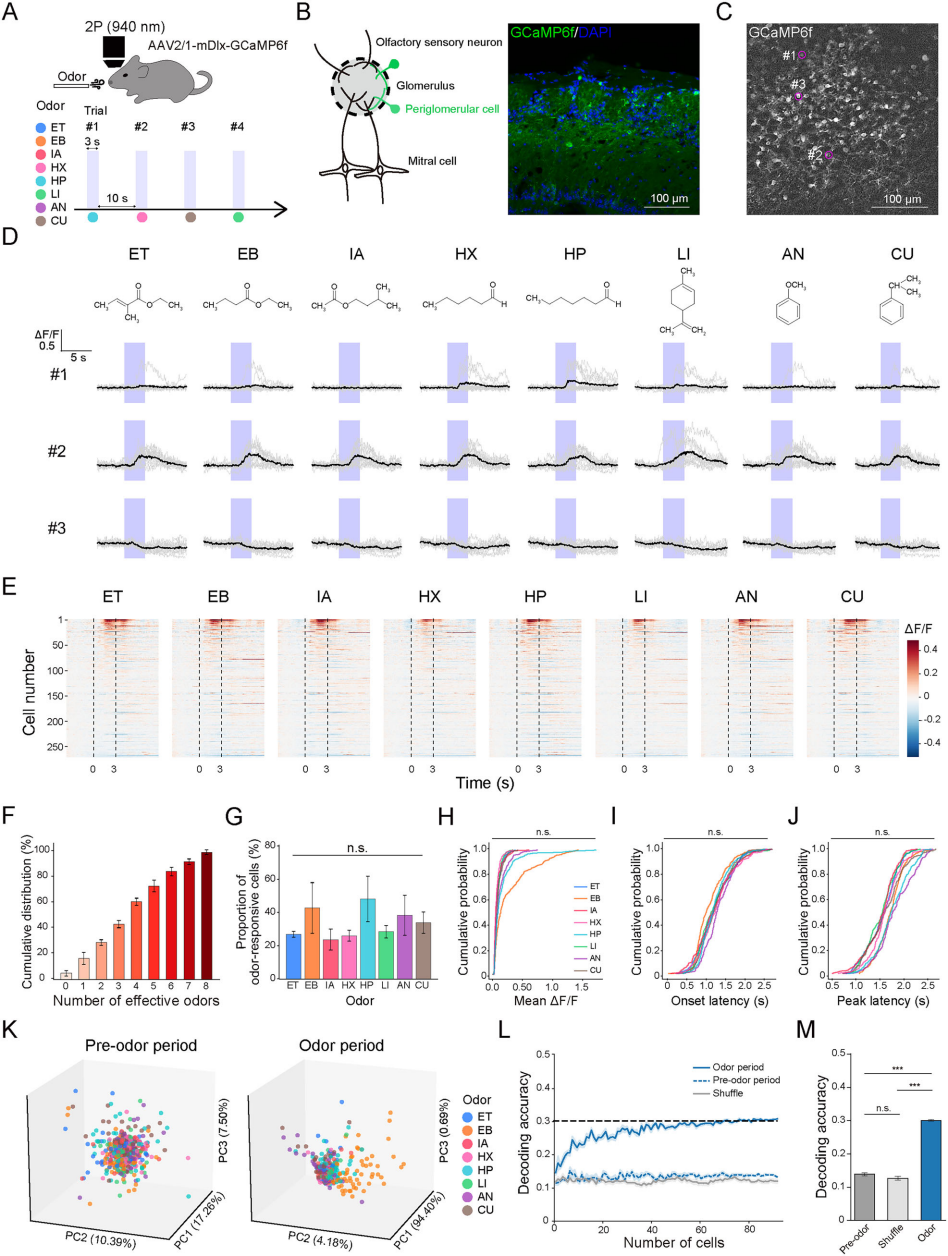
Odor representations in PGC population. ***A***, Experimental design of in vivo two-photon calcium imaging of head-fixed awake mice with odor stimulation. Mice were exposed to eight pure odorants (ET, ethyl tiglate; EB, ethyl butyrate; IA, isoamyl acetate; HX, hexanal; HP, heptanal; LI, (+)-limonene; AN, anisole; CU, cumene) in 15 randomized trials per odorant. ***B***, Coronal section of GCaMP6f fluorescence in the periglomerular layer in the OB. Scale bar, 100 µm. ***C***, In vivo two-photon fluorescence image of GCaMP6f-expressing PGCs in the OB. Cells #1–#3 are highlighted. Scale bar, 100 µm. ***D***, Representative odor-evoked calcium transients from Cells #1–#3 in ***C***. Gray lines indicate single trials, and black lines indicate trial averages. Blue-shaded area indicates the 3 s odor period. ***E***, Trial-averaged fluorescence changes of individual PGCs in each odorant. Cells are sorted according to their response amplitude during the odor period of ET, and the same order is maintained for other odorants. *n* = 268 cells from one mouse. ***F***, Cumulative distribution of the number of odorants that showed responses in individual PGCs. *n* = 821 cells from four mice. ***G***, Proportion of odor-responsive PGCs for each of eight odorants. *n* = 821 cells from four mice. One-way repeated–measures ANOVA on mouse-wide proportions (*F*_(7,21)_ = 1.119; *p* *=* 0.388). ***H–J***, Cumulative distribution of response amplitude (***H***), response onset latency (***I***), and peak latency (***J***). *n* = 216 (ET), 300 (EB), 217 (IA), 208 (HX), 328 (HP), 213(LI), 252 (AN), and 249 (CU) responsive cells from four mice. Repeated-measures ANOVA followed by Holm-corrected post hoc tests (mean Δ*F*/*F*, *F*_(7,21)_ = 1.170; *p* *=* 0.361; onset latency, *F*_(7,21)_ = 0.796; *p* *=* 0.599; peak latency, *F*_(7,21)_ = 1.392; *p* *=* 0.260). ***K***, PCA of population activity of odor-responsive PGCs. PC1, PC2, and PC3 explained 17.26, 10.39, and 7.50% in the preodor period and 94.40, 4.18, and 0.69% in the odor period. ***L***, Decoding accuracy plotted as a function of the number of cells for classifier training. Blue solid, blue dashed, and gray lines indicate the odor period, preodor period, and shuffled data, respectively. Shaded areas indicate mean ± SEM. Across repeats of fivefold cross-validation. Solid lines denote mean accuracy across 10 iterations. The horizontal black dashed line marks the accuracy threshold. *n* = 821 cells from four mice. ***M***, Decoding accuracy for the odor period, preodor period, and shuffled data when using 14 cells, the number at which decoding accuracy first exceeded the threshold in ***L***. *n* = 821 cells from four mice. One-way ANOVA followed by Holm-corrected post hoc tests. Shuffle versus preodor, *p* *=* 0.082; shuffle versus odor, *p* < 0.001; preodor versus odor, *p* < 0.001; all shaded areas and error bars denote the SEM. n.s., not significant; ***p < 0.001. Additional analyses of inhibitory odor responses and the relationships between odor selectivity and response properties are shown in Extended Data Figure 1-1. Detailed statistical analyses for Figure 1 and Extended Data Figure 1-1 are provided in Extended Data Figure 1-2.

**Figure 2.**
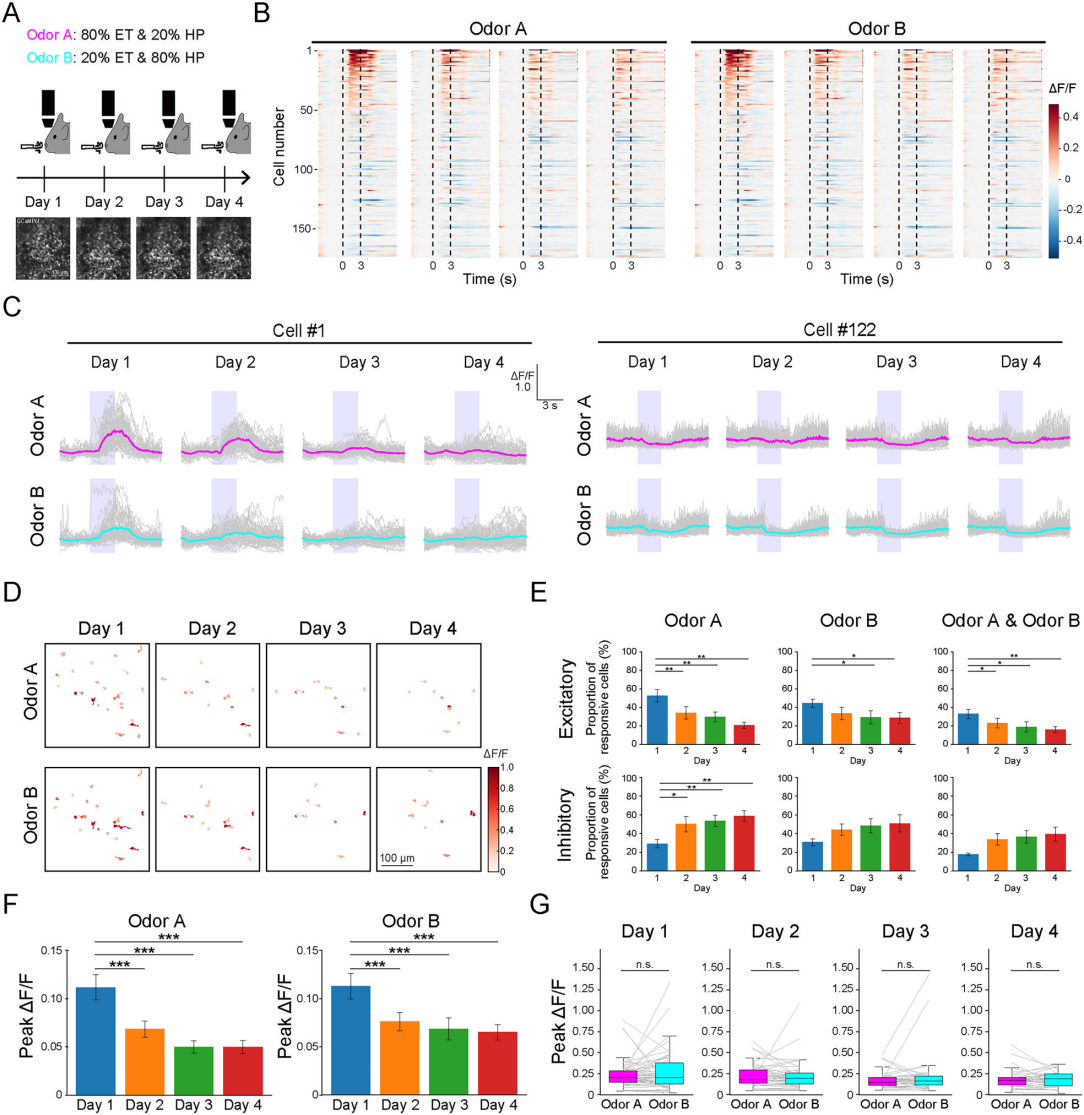
Population dynamics of PGCs during repeated passive odor exposure. ***A***, Experimental design for chronic in vivo two-photon calcium imaging of head-fixed awake mice during repeated passive odor exposure. Mice were presented with two odor mixtures (Odor A, 80% ET and 20% HP; Odor B, 20% ET and 80% HP). The same field of view was imaged across 4 consecutive days. Scale bar, 100 µm. ***B***, Trial-averaged fluorescence changes of individual PGCs to Odor A (top) and Odor B (bottom) across 4 d. Cells are sorted according to their response amplitude on Day 1, and the same order is maintained for Days 2–4. *n* = 172 cells from five mice. ***C***, Representative fluorescence traces of individual PGCs across Day 1–4 in response to Odor A and Odor B (Cell #1 and #122 in ***B***). Gray lines indicate single trials, and colored lines indicate trial averages. Blue-shaded area indicates the 3 s odor period. *n* = 172 cells from five mice. ***D***, Representative spatial distribution of odor-responsive PGCs and their amplitude across 4 d. Scale bar, 100 μm. ***E***, Proportions of PGCs showing excitatory or inhibitory responses selective for Odor A, Odor B, or both odors across the 4 d. *n* = 1036 (Day 1), 1011 (Day 2), 932 (Day 3), 838 (Day 4) cells from five mice. Repeated-measures one–way ANOVA followed by Holm-corrected post hoc tests. Excitatory Odor A, Day 1 versus Day 2, *p_adj_* *=* 0.007; Day 1 versus Day 3, *p_*adj*_* *=* 0.006; Day 1 versus Day 4, *p_*adj*_* *=* 0.004; Excitatory Odor B, Day 1 versus Day 3, *p_*adj*_ =* 0.028; Day 1 versus Day 4, *p_*adj*_ =* 0.046; Excitatory Odor A and Odor B, Day 1 versus Day 2, *p_*adj*_* *=* 0.043; Day 1 versus Day 3, *p_*adj* _=* 0.030; Day 1 versus Day 4, *p*_*adj* _*=* 0.008; Inhibitory Odor A, Day 1 versus Day 2, *p_adj_* *=* 0.013; Day 1 versus Day 3, p_*adj* _*=* 0.003; Day 1 versus Day 4, *p_adj_* *=* 0.003. Note that only significant post hoc comparisons are indicated. ***F***, Peak response amplitude across the 4 d for Odor A and Odor B. *n* = 172 (Days 1–4) cells from five mice. Repeated-measures ANOVA followed by Holm-corrected post hoc tests. Odor A, Day 1 versus Day 2, *p_*adj* _*< 0.001; Day 1 versus Day 3, *p_adj_* < 0.001; Day 1 versus Day 4, *p_*adj*_* < 0.001; Odor B, Day 1 versus Day 2, *p_*adj*_* < 0.001; Day 1 versus Day 3, *p_*adj*_* < 0.001; Day 1 versus Day 4, *p_*adj* _*< 0.001. Error bars denote the SEM. ***G***, Peak response amplitude of individual PGCs to Odor A and Odor B across 4 d. Gray lines connect the same cells across the two odors. *n* = 49 (Day 1), 42 (Day 2), 37 (Day 3), and 32 (Day 4) cells from five mice. Two-sided paired *t* test. Day 1, *p* = 0.307; Day 2, *p* = 0.960; Day 3, *p* = 0.198; Day 4, *p* = 0.696. n.s., not significant; *p < 0.05; **p < 0.01; ***p < 0.001. Spatial distributions of excitatory- and inhibitory-responsive PGCs during repeated passive odor exposure are shown in Extended Data Figure 2-1. Detailed statistical analyses for Figure 2 and Extended Data Figure 2-1 are provided in Extended Data Figure 2-2.

A custom-built olfactometer was used to deliver odor stimuli through solenoid valves controlled by the LabVIEW software (National Instruments) as previously described (Chu et al., 2016; Li et al., 2018). Airflow was adjusted at 1.0 L/min. For the passive odor exposure experiment (Fig. 1), the following odorants were used: ethyl tiglate (ET; Tokyo Chemical Industry), ethyl butyrate (EB; Tokyo Chemical Industry), isoamyl acetate (IA; Tokyo Chemical Industry), hexanal (HX; Tokyo Chemical Industry), heptanal (HP; Tokyo Chemical Industry), (+)-limonene (LI; Tokyo Chemical Industry), anisole (AN; Tokyo Chemical Industry), and cumene (CU; Tokyo Chemical Industry). For the repeated passive odor exposure experiment (Fig. 2) and Go/No-go odor discrimination task (Figs. 4 and 5), a mixture of ET and HP was used. All odorants were diluted 1:10 (v/v) in mineral oil (Nacalai Tesque) before use.

### Passive odor exposure test

Mice were water restricted starting 1 week after cranial window implantation and were given ∼1 ml of water daily to maintain their BW at 80–85% of the initial value. Head-fixed mice were presented with odors under the two-photon microscope.

For the passive odor exposure test (Fig. 1), each of the eight pure odorants (ET, EB, IA, HX, HP, LI, AN, CU) was presented 15 times in a randomized order. Each trial consisted of a 3 s odor presentation followed by a 10 s intertrial interval (ITI). For the repeated passive odor exposure experiment (Fig. 2), mice were repeatedly exposed to two odor mixtures for 4 consecutive days. Each odor mixture (Odor A, 80% ET and 20% HP; Odor B, 20% ET and 80% HP) was presented 50 times per day, and each trial consisted of a 3 s odor presentation followed by a 12 s ITI.

### Go/No-go odor discrimination task

**Figure 3.**
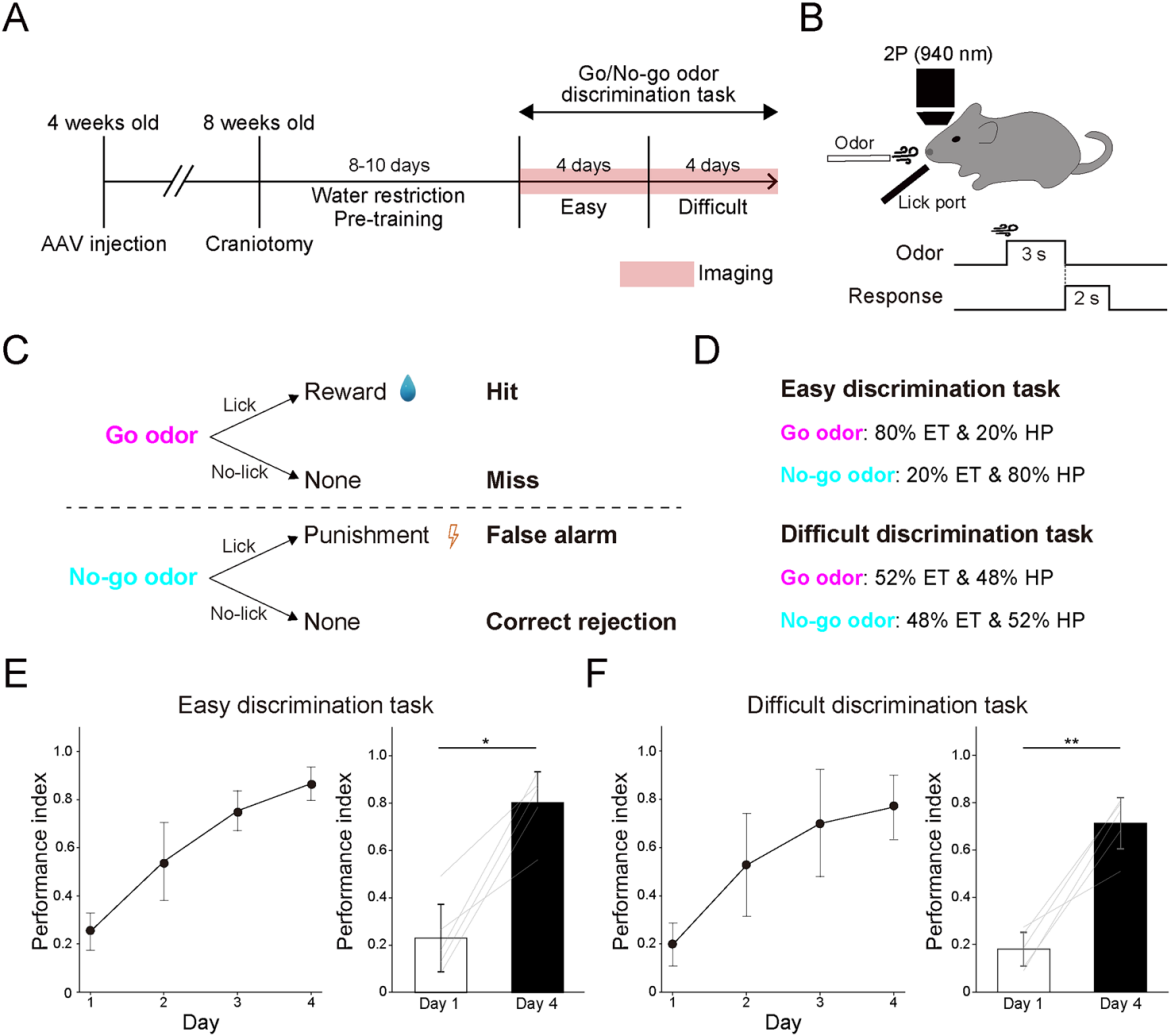
Go/No-go odor discrimination task. ***A***, Experimental timeline of the Go/No-go odor discrimination task. ***B***, Schematic of the behavioral paradigm. Each trial consisted of a 3 s odor presentation followed by a 2 s response window. ***C***, Task schematics. Licking in a Go odor trial was rewarded with water (hit), while failure to lick was unrewarded (miss). Licking in a No-go trial was punished (false alarm), while withholding licking was correct (correct rejection). ***D***, Odor compositions for easy and difficult discrimination tasks. Easy discrimination task: Go odor, 80% ET and 20% HP; No-go odor, 20% ET and 80% HP. Difficult discrimination task: Go odor, 52% ET and 48% HP; No-go odor, 48% ET and 52% HP. ***E***, Learning curve of the easy discrimination task (left) and performance comparison between the first and the last day (right). Gray lines indicate the performance index of individual mice between Day 1 and Day 4. *n* = 5 mice. Two-sided paired *t* test. *p* *=* 0.014. ***F***, Learning curve of the difficult discrimination task (left) and performance comparison between the first and the last day (right). Gray lines indicate the performance index of each mouse on the first and last days. *n* = 5 mice. Two-sided paired *t* test. *p* *=* 0.002. All error bars denote the SEM. **p < 0.01. Detailed statistical analyses are provided in Extended Data Figure 3-1.

**Figure 4.**
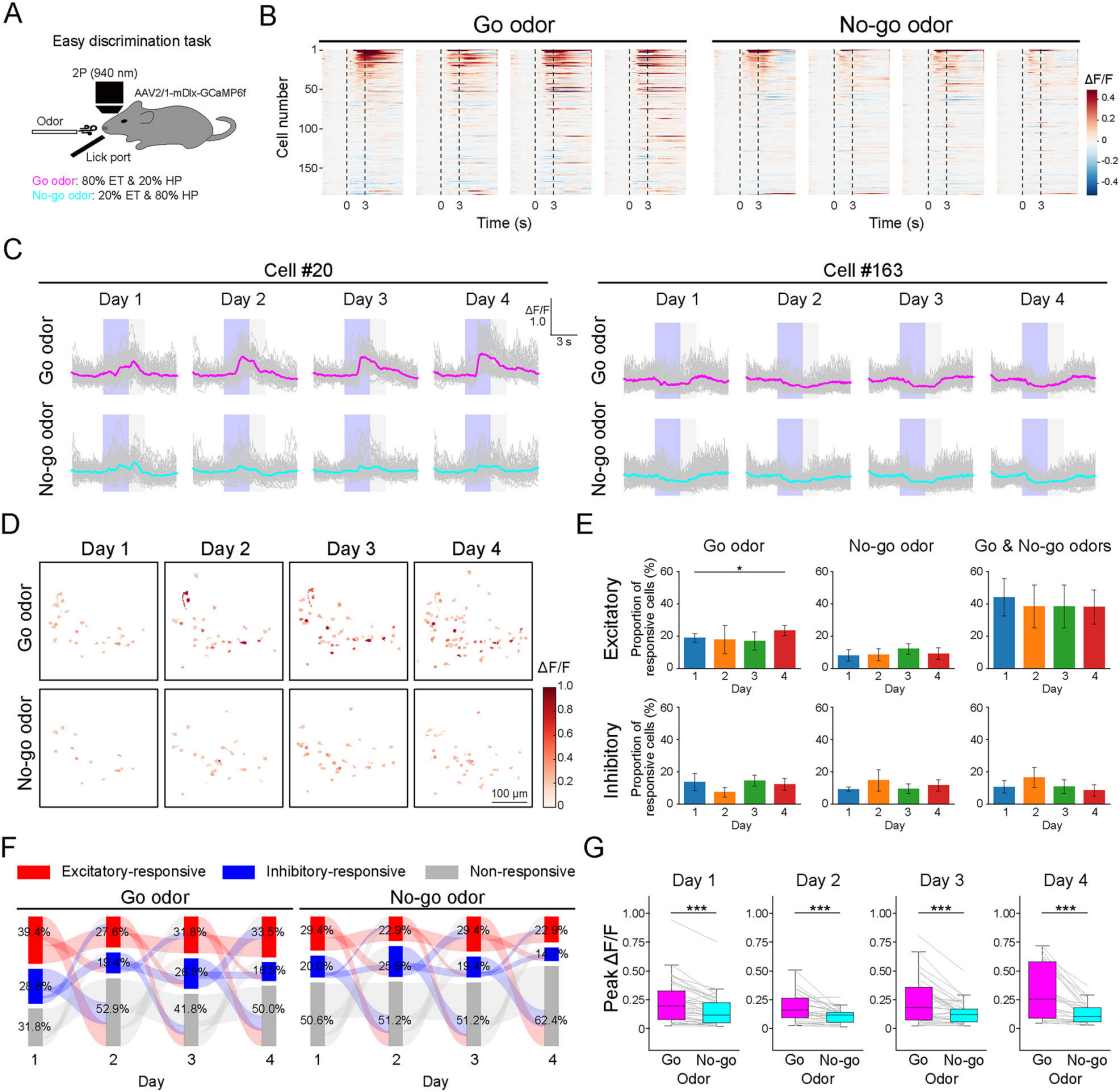
Reorganization of PGC responses during Go/No-go easy odor discrimination task. ***A***, Experimental design of in vivo two-photon calcium imaging of head-fixed awake mice with the easy discrimination task. Go odor, 80% ET and 20% HP; No-go odor, 20% ET and 80% HP. ***B***, Trial-averaged fluorescence changes of individual PGCs to Go odor (left) and No-go odor (right) across 4 d. Cells are sorted according to their response amplitude on Day 1, and the same order is maintained for Days 2–4. *n* = 180 cells from five mice. ***C***, Representative fluorescence traces of individual PGCs (Cell #20 and #163 in ***B***). Gray lines indicate single trials, and colored lines indicate trial averages. Blue- and gray-shaded areas indicate the odor and response periods, respectively. ***D***, Representative spatial distribution of odor-responsive PGCs and their amplitude across 4 d. Scale bar, 100 µm. ***E***, Proportions of PGCs showing excitatory or inhibitory responses selective for Go odor, No-go odor, or both odors across the 4 training days. *n* = 843 (Day 1), 873 (Day 2), 917 (Day 3), and 902 (Day 4) cells from five mice. Repeated-measures ANOVA followed by Holm-corrected post hoc tests. Excitatory Go odor, Day 1 versus Day 4, *p_*adj*_* *=* 0.019. Note that only significant post hoc comparisons are indicated. ***F***, Alluvial diagrams illustrating phenotype transitions of individual PGCs. Red, blue, and gray ribbons represent excitatory, inhibitory, and nonresponsive cells, respectively. Numbers indicate the percentage of cells in each category. *n* = 180 cells from five mice. ***G***, Peak response amplitude of individual PGCs to Go odor and No-go odor across 4 d. Gray lines connect the same cells across the two odors. *n* = 39 (Day 1), 30 (Day 2), 34 (Day 3), and 28 (Day 4) cells from five mice. Two-sided paired *t* test. Day 1, *p* < 0.001; Day 2, *p* < 0.001; Day 3, *p* < 0.001; Day 4, *p* < 0.001. Error bars denote the SEM. n.s., not significant; *p < 0.05; **p < 0.01; ***p < 0.001. Spatial distributions of excitatory- and inhibitory-responsive PGCs during easy discrimination task are shown in Extended Data Figure 4-1. Detailed statistical analyses for Figure 4 and Extended Data Figure 4-1 are provided in Extended Data Figure 4-2.

**Figure 5.**
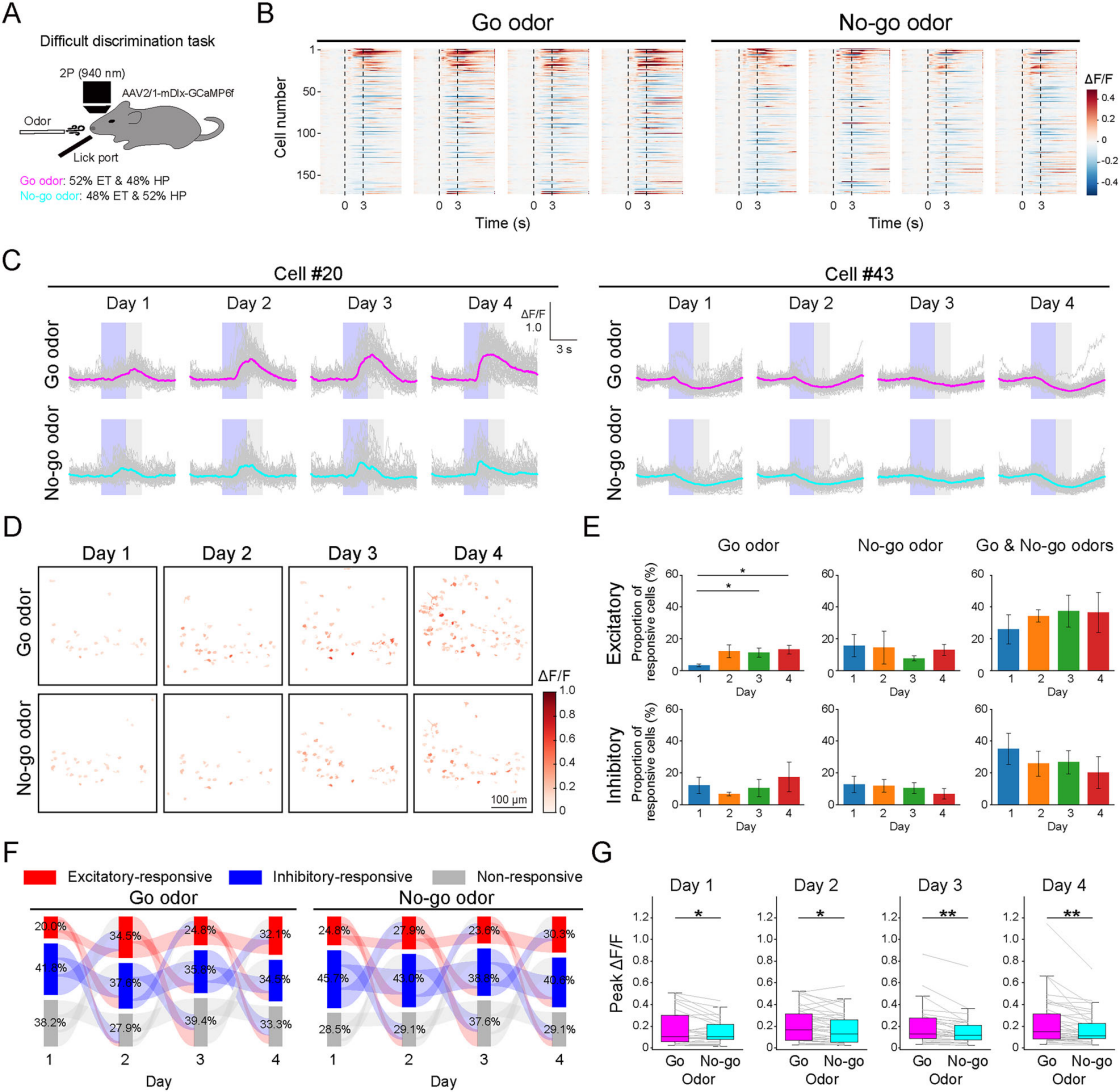
Reorganization of PGC responses during Go/No-go difficult odor discrimination task. ***A***, Experimental design of in vivo two-photon calcium imaging of head-fixed awake mice with the difficult discrimination task. Go odor, 52% ET and 48% HP; No-go odor, 48% ET and 52% HP. ***B***, Trial-averaged fluorescence changes of individual PGCs to Go odor (left) and No-go odor (right) across 4 d. Cells are sorted according to their response amplitude on Day 1, and the same order is maintained for Days 2–4. *n* = 174 cells from five mice. ***C***, Representative fluorescence traces of individual PGCs (Cell #20 and #43 in ***B***). Gray lines indicate single trials, and colored lines indicate trial averages. Blue- and gray-shaded areas indicate the odor and response periods, respectively. ***D***, Representative spatial distribution of odor-responsive PGCs and their amplitude across 4 d. Scale bar, 100 µm. ***E***, Proportions of PGCs showing excitatory or inhibitory responses selective for Go odor, No-go odor, or both odors across the 4 training days. *n* = 927 (Day 1), 999 (Day 2), 994 (Day 3), and 1,023 (Day 4) cells from five mice. Repeated-measures ANOVA followed by Holm-corrected post hoc tests. Excitatory Go odor, Day 1 versus Day 3, *p_adj_* = 0.045, Day 1 versus Day 4, *p_*adj* _=* 0.030. Note that only significant post hoc comparisons are indicated. ***F***, Alluvial diagrams illustrating phenotype transitions of individual PGCs. Red, blue, and gray ribbons represent excitatory, inhibitory, and nonresponsive cells, respectively. Numbers indicate the percentage of cells in each category. *n* = 174 cells from five mice. ***G***, Peak response amplitude of individual PGCs to Go odor and No-go odor across 4 d. Gray lines connect the same cells across the two odors. *n* = 28 (Day 1), 41 (Day 2), 31 (Day 3), and 38 (Day 4) cells from five mice. Two-sided paired *t* test. Day 1, *p* *=* 0.035; Day 2, *p* *=* 0.014; Day 3, *p* *=* 0.001; Day 4, *p* *=* 0.002. Error bars denote the SEM. n.s., not significant; *p < 0.05; **p < 0.01; ***p < 0.001. Spatial distributions of excitatory- and inhibitory-responsive PGCs during difficult discrimination task are shown in Extended Data Figure 5-1. Detailed statistical analyses for Figure 5 and Extended Data Figure 5-1 are provided in Extended Data Figure 5-2.

Mice were trained to discriminate two different odor mixtures in a Go/No-go paradigm under head-fixed conditions (Figs. 3–5). Mice were water restricted starting 1 week after cranial window implantation and were given ∼1 ml of water daily to maintain their BW at 80–85% of the initial value. Then, mice were subjected to a pretraining phase followed by a training phase.

The pretraining phase started 1 week after water restriction. Mice were initially habituated to be head-fixed for 30 min under the two-photon microscope where the odor discrimination task was conducted. On the following day, mice were rewarded for licking the lick port without any odor stimuli. Then, mice were trained with a pure odorant (AN) with a 2 s ITI. Once mice reached a success rate above 80%, the ITI was incrementally extended by 2 s every 20 trials until it reached 12 s. When the performance exceeded 80% at a 12 s ITI, mice proceeded to the training phase.

During the training phase, mice performed one session per day, which consisted of 100 trials. Each trial included a 3 s odor period, a 2 s response period, and a 12 s ITI. Go odor and No-go odor were randomly delivered with no more than three consecutive trials of the same odor. Water (5 μl) was delivered as a reward when the mouse licked the lick port at least once during the response period. Licking during the No-go odor period was punished with high-frequency white noise. During the odor discrimination, daily water supplementation was performed to keep BW at 85% of the original value before mice were kept in their home cages where food was available *ad libitum*. Behavioral performance was calculated as performance index (PI), PI = hits/(hits + misses) − false alarms/(false alarms + correct rejections). Under this definition, the theoretical chance level of PI is 0 because random licking yields equal hit and false alarm rates. Mice were trained in the easy discrimination task until PI exceeded 0.75 and then proceeded to the difficult discrimination task. Both Go and No-go odors were binary mixtures of ET and HP with different ratios. For the easy discrimination task, the Go odor was 80% ET and 20% HP, while the No-go odor was 20% ET and 80% HP. For the difficult discrimination task, the Go odor was 52% ET and 48% HP, while the No-go odor was 48% ET and 52% HP.

We used the same group of mice across all task sessions, including the easy and difficult discrimination tasks, and imaged the same field of view within each mouse throughout the task series. Within each task, we tracked the same set of cells across training days.

### Tissue preparation

Tissue preparation was performed as described previously (Sakamoto et al., 2022). Mice were deeply anesthetized with the anesthetic mixture described above and transcardially perfused with 25 ml of PBS and 50 ml of 4% PFA/PB, pH 7.4. Brains were postfixed in the perfusing solution overnight at 4°C and then immersed for 24 h in 30% sucrose in PBS. Brains were embedded in OCT compound (Sakura Finetek Japan) and frozen at −80°C.

Tissue blocks were cut into 30-μm-thick slices with a cryostat (CM1950, Leica) and floated in PBS. For nuclear staining, brain slices were incubated with DAPI for 10 min at room temperature before mounting on slides. Imaging was performed using a confocal laser-scanning microscope (LSM 880, Carl Zeiss) with a 20× objective lens and 405 and 488 nm lasers.

### Data analysis methods

#### Image analysis

Image analysis was performed using ImageJ/Fiji (NIH), MATLAB (MathWorks), and Python (https://www.python.org/). Motion correction was performed by the Suite2P toolbox (https://github.com/MouseLand/suite2p/; Pachitariu et al., 2017). Semiautomatic segmentation was performed in the motion-corrected data using the Suite2P toolbox. To track the same PGCs across days, we used the ROIMatchPub toolbox (https://github.com/ransona/ROIMatchPub). The relative fluorescence change was calculated as Δ*F*/*F* = (*F* − *F*_0_) / *F*_0_, where *F* represents the fluorescence intensity at any time point and *F*_0_ is the baseline fluorescence signal averaged over a 3 s period before the onset of odor stimulation (preodor period).

#### Quantification of calcium response amplitude and temporal features

Response magnitude and temporal features were quantified from single-trial Δ*F*/*F* traces of each cell. Δ*F*/*F* signals were segmented into trials, and analyses were performed using the preodor (3 s before odor onset), the odor period (3 s from odor onset), and postodor period (3 s after odor offset). The mean response amplitude was calculated by averaging Δ*F*/*F* values within the odor period for each trial and then averaging across trials for each cell. Peak amplitude was defined as the maximum Δ*F*/*F* within each trial. Peak latency was defined as the time from odor onset to this peak Δ*F*/*F*. Onset latency was defined as the time from odor onset to the first significant increase in Δ*F*/*F*. For each trial, a response threshold was defined as the baseline mean +1.5 standard deviation (SD) of the Δ*F*/*F* values during the preodor period. Within the odor period, the onset time was identified as the first time point at which the Δ*F*/*F* exceeded this threshold for at least 0.2 s. Trials without detectable threshold crossing were excluded from the calculation. All metrics were computed on a per-trial basis and subsequently averaged across trials for each cell.

#### Identification of odor-responsive PGCs

Odor-responsive PGCs were identified using the following criteria as previously described with minor modifications (Chu et al., 2016). For each cell, the mean ΔF/F values during the preodor period and the odor period were calculated for each trial, and these trial-to-trial means were compared across trials using a Wilcoxon signed-rank test. Cells were classified as excitatory-responsive cells when the odor period activity was significantly greater than the preodor period activity (one-sided test, *p* < 0.05) and as inhibitory-responsive cells when the odor period activity was significantly lower than the preodor period activity (one-sided test, *p* < 0.05). Cells that met neither criterion were classified as nonresponsive.

#### Analysis of spatial distribution of PGC responses

Pairwise distances between neurons were calculated based on their spatial positions within the imaging field of view. Neurons were classified as excitatory-responsive or inhibitory-responsive as described above, and the Euclidean distance was computed for all pairs of neurons within each group using the centroid coordinates of each region of interest (ROI). The centroid of each ROI was determined from the Suite2P segmentation. For each mouse, the cumulative distribution of pairwise distances was computed, and the mean cumulative distribution across mice was plotted. For statistical comparison, the median pairwise distance was calculated for each mouse, and the median values were compared between excitatory-responsive and inhibitory-responsive populations using a paired Wilcoxon signed-rank test (two-sided).

#### Principal component analysis

To quantify how the PGC population activity diverges among the task events within and between learning days, we performed principal component analysis (PCA) on the Δ*F*/*F* data matrix of each mouse. The unique trajectory of each task event in learning Day 1 and Day 4 was determined in the principal component space; then the frame-by-frame Euclidean distances between the trajectories were calculated. In the passive odor exposure test, the size of the row and column of the data matrix was the number of successive frames in the event (45) and the product of the number of PGCs (N*; see below) and the number of odors (8), respectively, and each element of this matrix was the trial-averaged Δ*F*/*F* in a PGC in a frame given an odor. The distance between the trajectories of Odors A and B was compared. In the odor discrimination test, the size of the row and column of the data matrix was the number of PGCs and the product of the number of event frames (198) and the number of odors (2), respectively. The Euclidean distance between the Go and No-go trajectories was computed. The PCA analysis above was implemented in Python with the scikit-learn library (https://scikit-learn.org/stable/).

#### Decoding analysis and cell-type–specific contributions across learning

For the decoding analysis, we used Linear Support Vector Classifier (Linear SVC; scikit-learn; https://scikit-learn.org/stable/). For each mouse and task condition, we constructed trial-wise population vectors from the mean Δ*F*/*F* within two fixed 3 s time windows corresponding to the preodor and odor periods. Decoding accuracy was evaluated at the trial level using fivefold cross-validation, with 80% of trials used for training and 20% for testing in each fold. To examine how decoding accuracy depends on population size (*N*), we randomly sampled *N* cells*,* varying *N* from one up to the total number of recorded cells for each mouse and task condition, and then trained the Linear SVC for each *N* as described above. This subsampling procedure was repeated multiple times, and decoding accuracy for given *N* was calculated as the mean across the five folds. To assess whether the decoding accuracy exceeded the chance level, we also used shuffled data as a control. This shuffling procedure was performed across all paradigms, and results were consistent across conditions. Therefore, we show the result only in Figures 1, *K* and *L* to avoid redundancy.

**Figure 6.**
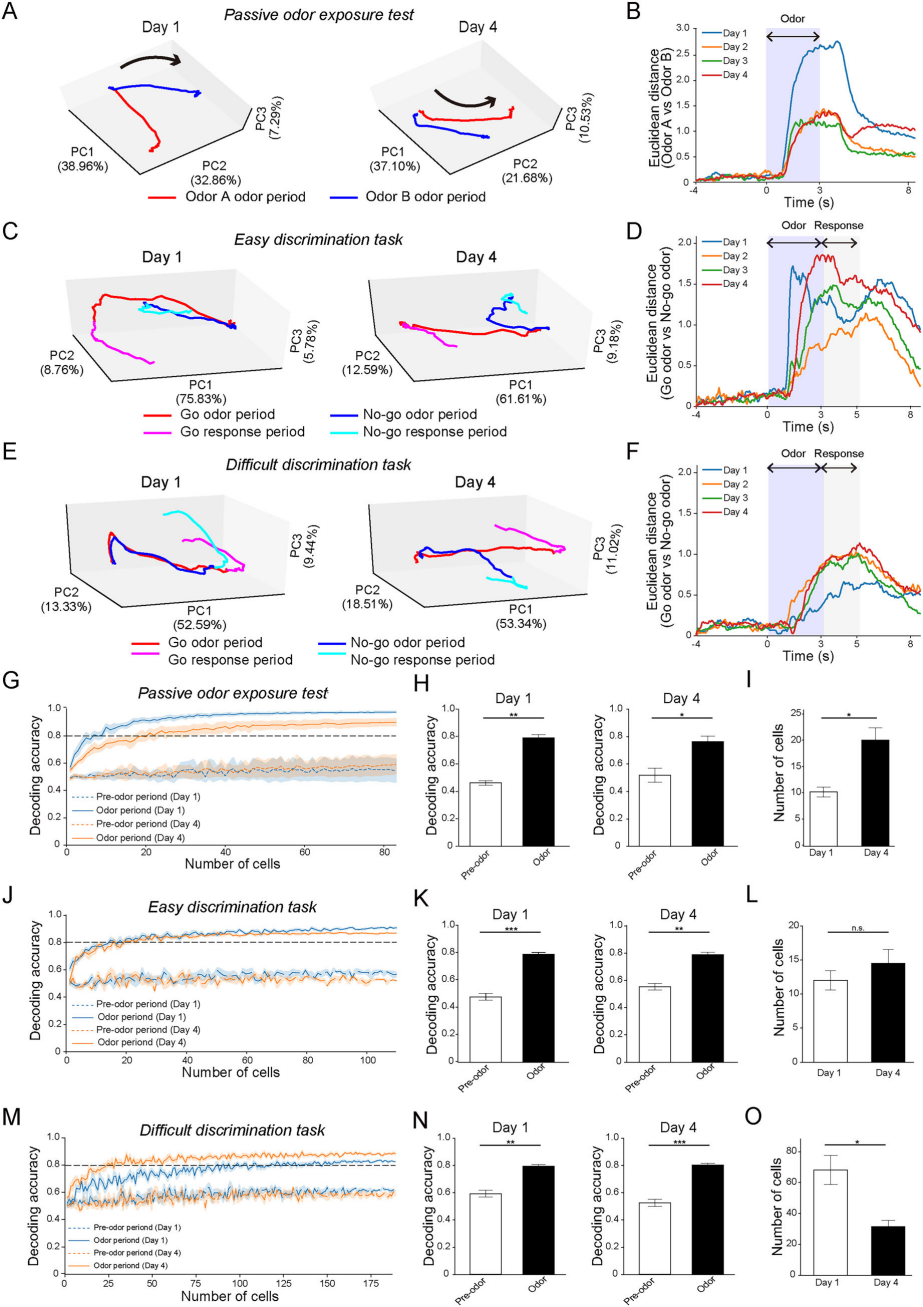
Comparison of population dynamics of PGCs across task conditions. ***A***, PCA trajectories of population responses to Odor A and Odor B on Day 1 and Day 4 during the repeated passive odor exposure condition. Each trajectory represents population activity during the odor periods. PC1, PC2, and PC3 explained 38.96, 32.86, and 7.29% on Day 1 and 37.10, 21.68, and 10.53% on Day 4. ***B***, Time course of the Euclidean distance between population trajectories for Odor A and Odor B. ***C***, PCA trajectories of population responses to Go odor and No-go odor on Day 1 and Day 4 during the easy discrimination task. Each trajectory represents the population activity during the preodor, odor, and response periods. PC1, PC2, and PC3 explained 75.83, 8.76, and 5.78% on Day 1 and 61.61, 12.59, and 9.18% on Day 4. ***D***, Time course of the Euclidean distance between population trajectories for Go odor and No-go odor. ***E***, PCA trajectories of population responses to Go odor and No-go odor on Day 1 and Day 4 during the difficult discrimination task. Each trajectory represents the population activity during the preodor, odor, and response periods. PC1, PC2, and PC3 explained 52.59, 13.33, and 9.44% on Day 1 and 53.34, 18.51, and 11.02% on Day 4. ***F***, Time course of the Euclidean distance between population trajectories for Go odor and No-go odor. ***G***, Decoding accuracy plotted as a function of the number of cells used for classifier training during the repeated passive odor exposure condition. The number of randomly selected cells was increased from 1 to the maximum number of cells common across all mice. Solid and dashed lines represent odor and preodor periods, respectively. Shaded areas indicate mean ± SEM. Across fivefold cross-validation. Solid lines denote mean accuracy across 10 iterations. The horizontal black dashed line marks the accuracy threshold. ***H***, Decoding accuracy during preodor and odor periods on Day 1 and Day 4 when using 19 cells, the number at which decoding accuracy first exceeded the threshold in the odor period in ***G***. Two-sided paired *t* test, *p* = 0.001 (Day 1) and *p* *=* 0.0149 (Day 4). ***I***, The number of cells required to reach the threshold of decoding accuracy on Day 1 and Day 4. *n* = 172 cells from five mice. Two-sided paired *t* test, *p* *=* 0.037. ***J***, Decoding accuracy plotted as a function of the number of cells used for classifier training during the easy discrimination task. Solid and dashed lines represent odor and preodor periods, respectively. Shaded areas indicate mean ± SEM. Across fivefold cross-validation. Solid lines denote mean accuracy across 10 iterations. The horizontal black dashed line marks the accuracy threshold. ***K***, Decoding accuracy during preodor and odor periods on Day 1 and Day 4 when using 15 cells, the number at which decoding accuracy first exceeded the threshold in the odor period in ***J***. Two-sided paired *t* test, *p* < 0.001 (Day 1) and *p* = 0.001 (Day 4). ***L***, The number of cells required to reach the threshold of decoding accuracy on Day 1 and Day 4. Two-sided paired *t* test, *p* *=* 0.239. ***M***, Decoding accuracy plotted as a function of the number of cells used for classifier training during the difficult discrimination task. Shaded areas indicate mean ± SEM. across fivefold cross-validation. Solid lines denote mean accuracy across 10 iterations. The horizontal black dashed line marks the accuracy threshold. ***N***, Decoding accuracy during preodor and odor periods on Day 1 and Day 4 when using 85 cells, the number at which decoding accuracy first exceeded the threshold in the odor period in ***M***. Two-sided paired *t* test, *p* *=* 0.002 (Day 1) and *p* < 0.001 (Day 4). ***O***, The number of cells required to reach the threshold of decoding accuracy on Day 1 and Day 4. Two-sided paired *t* test, *p* *=* 0.044. All shaded areas and error bars denote the SEM. n.s., not significant; *p < 0.05; **p < 0.01. The contributions of excitatory- and inhibitory-responsive PGCs to decoding accuracy during easy and difficult discrimination tasks are shown in Extended Data Figure 6-1. Detailed statistical analyses for Figure 6 and Extended Data Figure 6-1 are provided in Extended Data Figure 6-2.

We calculated the population size at which the decoding accuracy during the odor period first exceeded the threshold [0.30 for eight-class decoding (Fig. 1); 0.80 for two-class decoding (Fig. 6), then defined N* as the average of them over the repeats and the mice]. These thresholds were statistically validated to be significantly higher than the chance level (*p* < 0.05, one-sided binomial test). At N*, we extracted the full distributions of decoding accuracy scores (mean ± SEM) for both odor and preodor periods and compared them statistically.

To quantify the contribution of individual neurons to decoding performance, we used the absolute values of the linear classifier weights. For each cross-validation fold, feature weights obtained from the trained Linear SVC were extracted, and their absolute values were taken to reflect the magnitude of each neuron's contribution to classification. These values were then averaged across folds to obtain a stable estimate of contribution for each neuron. Subsequent analyses compared these contribution values across neuronal categories.

### Statistical analysis

All statistical analyses of the acquired data were performed with Python. For each figure, a statistical test matching the structure of the experiment and the structure of the data was employed. The specific test used for each comparison is indicated in the corresponding figure legend, including whether the test was one- or two-sided. When repeated-measures ANOVA was used, post hoc pairwise comparisons were performed with Holm correction for multiple comparisons. Adjusted *p* values after Holm correction are reported as *p_adj_* [*p < 0.05; **p < 0.01; ***p < 0.001; n.s., not significant (*p* > 0.05) for all statistical analyses presented in figures]. No statistical tests were performed to predetermine the sample size. Data acquisition and analysis were not performed blind to the conditions of the experiments. Experimental sample sizes are mentioned in the figure panels and legends. A complete summary of all statistical tests, including sample size, degrees of freedom, exact *p* value, correction method, and post hoc comparisons, is provided in Extended Data Figures 1-2, 2-2, 3-1, 4-2, 5-2, and 6-2.

## Results

### Odor representations in PGC population

To investigate how PGCs represent odor information, we performed in vivo two-photon calcium imaging to record the activity of PGCs in the dorsal region of the OB. Head-fixed awake mice were presented with a series of eight pure odorants randomly for 3 s each (15 trials per odor). To express the genetically encoded calcium indicator GCaMP6f in the PGCs, we injected an AAV encoding GCaMP6f under the control of the mDlx enhancer, which drives expression in GABAergic inhibitory neurons of the forebrain (Dimidschstein et al., 2016; Fig. 1*A*). Four weeks after viral injection, robust GCaMP6f expression was observed in PGCs around the injection site (Fig. 1*B*). In vivo two-photon imaging was conducted at depths of 100–150 µm from the pial surface, corresponding to the periglomerular layer (Fig. 1*C*).

10.1523/ENEURO.0171-26.2026.f1-1 Figure 1-1 **Population activity dynamics and variability**. **A**, Proportion of PGCs showing inhibitory responses to each odorant. n = 821 cells from 4 mice. Repeated-measures ANOVA, F (7, 21) = 0.599, *p* = 0.749. **B–D**, Relationship between the number of responsive odors per neuron and peak response amplitude (B), response onset latency (C), and peak latency (D). Each dot represents an individual neuron. Spearman’s rank correlation coefficients (ρ) and corresponding p-values are indicated in each panel. Error bars denote the s.e.m. n.s., not significant. Detailed statistical analyses are provided in Figure 1-2. Download Figure 1-1, TIF file.

10.1523/ENEURO.0171-26.2026.f1-2 Figure 1-2 **Statistical summary for Figures 1 and 1-1.** Summary of the sample size, statistical test, degrees of freedom, exact *p*-values, and multiple comparisons for the analyses shown in Figures 1 and 1-1. Download Figure 1-2, DOCX file.

Two-photon imaging revealed heterogeneous odor-evoked responses among individual PGCs. Some neurons responded broadly to multiple odors, whereas others exhibited high selectivity (Fig. 1*D*,*E*). To determine odor responsiveness, cells were classified as excitatory-responsive when the odor period activity was significantly greater than the preodor period activity and as inhibitory-responsive when the odor period activity was significantly lower (see Materials and Methods). Response patterns across all recorded PGCs showed that the populations of odor-responsive PGCs mostly overlapped across odorants, indicating that PGCs display low selectivity across odors with different chemical structures (Fig. 1*E*). Quantitative analysis confirmed that the majority of odor-responsive PGCs were activated by multiple odorants (Fig. 1*F*). In addition, the proportion of odor-responsive PGCs responding to each odorant, response amplitude, onset latency, and peak latency were comparable across odorants, indicating no bias toward specific odor identities (Fig. 1*G*–*J*). We note that not only excitatory but also inhibitory responses to each odorant were observed (Fig. 1*H*; Extended Data Fig. 1-1*A*). We also found no significant correlation between odor selectivity and response kinetics or sensitivity (Extended Data Fig. 1-1*B*–*D*).

To explore the odor representations at the population level, we applied PCA to fluorescence signals of odor-responsive PGCs. During odor presentation, the activities of odor-responsive PGCs became more clustered in the principal component space compared with those during the preodor period, suggesting that the population responses became more odor-specific (Fig. 1*K*). To further assess whether PGC population activity contains information to discriminate odor identity, we trained a Linear Support Vector Classifier (SVC) on ensemble PGC activity. Decoding accuracy improved progressively with the number of cells used for training, reaching ∼30% with ∼20 neurons during the odor period, whereas preodor period accuracy remained low regardless of population size (Fig. 1*L*). At the threshold population size (14 cells), odor period accuracy was significantly higher than both the preodor period accuracy and the accuracy from the shuffled data, whereas preodor period accuracy did not differ significantly from the shuffled control (Fig. 1*M*). These results demonstrate that although the odor-responsive PGC population is relatively small, ensemble activity during odor presentation contains sufficient information to encode odor identity in the OB. For reference, detailed statistical analyses for Figure 1 and Extended Data Figure 1-1 are provided in Extended Data Figure 1-2.

### Repeated passive odor exposure reduces the number of responsive PGCs but maintains odor response properties

It is well known that sensory representations can be modified through sensory experience (Feldman, 2000; Kato et al., 2012, 2015; Abraham et al., 2014; Chu et al., 2016; Bauer et al., 2024). To examine whether prolonged odor exposure alters PGC activity in the OB, mice were presented with binary odor mixtures of ET and HP at different ratios (Odor A, 80% ET and 20% HP; Odor B, 20% ET and 80% HP) for 4 consecutive days (Fig. 2*A*). We repeatedly imaged the same population of PGCs across days.

10.1523/ENEURO.0171-26.2026.f2-1 Figure 2-1 **Spatial organization of excitatory- and inhibitory-responsive PGCs during repeated passive odor exposure**. Cumulative distributions of pairwise distances among excitatory (red) and inhibitory (blue) cells across Days 1–4 during repeated passive odor exposure. Statistical comparisons between excitatory and inhibitory populations were performed using the Wilcoxon signed-rank test. Day 1, Odor A, *p* = 0.138, excitatory *n* = 543 cells, inhibitory *n* = 303 cells; Odor B, *p* = 0.500, excitatory *n* = 478 cells, inhibitory *n* = 303 cells; Day 2, Odor A, *p* = 0.685, excitatory *n* = 303 cells, inhibitory *n* = 555 cells; Odor B, *p* = 0.892, excitatory *n* = 304 cells, inhibitory *n* = 458 cells; Day 3, Odor A, *p* = 0.079, excitatory *n* = 238 cells, inhibitory *n* = 544 cells; Odor B, *p* = 0.685, excitatory *n* = 260 cells, inhibitory *n* = 454 cells; Day 4, Odor A, *p* = 0.138, excitatory *n* = 162 cells, inhibitory *n* = 521 cells; Odor B, *p* = 0.892, excitatory *n* = 229 cells, inhibitory *n* = 422 cells, from 5 mice. Detailed statistical analyses are provided in Figure 2-2. Download Figure 2-1, TIF file.

10.1523/ENEURO.0171-26.2026.f2-2 Figure 2-2 **Statistical summary for Figures 2 and 2-1.** Summary of the sample size, statistical test, degrees of freedom, exact *p*-values, and multiple comparisons for the analyses shown in Figures 2 and 2-1. Download Figure 2-2, DOCX file.

Although odor-evoked responses in individual PGCs were largely stable, their response amplitudes gradually weakened with repeated odor exposure (Fig. 2*B*,*C*). This gradual attenuation suggests a progressive adaptation of PGC activity to repeated sensory input. Spatial activity maps of PGCs showed a gradual reduction in both the number of responsive cells and the response amplitude across days (Fig. 2*D*). Thus, the reduction in responsiveness occurred broadly across the field of view rather than being restricted to a spatially segregated subset of PGCs. There was no obvious spatial clustering of Odor A- and Odor B-responsive cells in the imaging field, indicating that the attenuation reflects a reduction in global PGC activity rather than a localized change in restricted glomeruli (Extended Data Fig. 2-1). To quantify these changes, we classified individual neurons based on their response profiles into excitatory or inhibitory categories for Odor A, Odor B, or both odors. For this analysis, cells were identified as excitatory- or inhibitory-responsive when odor period activity was significantly higher or lower than preodor activity, respectively (one-sided Wilcoxon signed-rank test, *p* < 0.05; see Materials and Methods).We found that the fraction of PGCs exhibiting excitatory responses to either odor mixture progressively declined across days, whereas the proportion of PGCs showing inhibitory responses tended to increase (Fig. 2*E*). Furthermore, the peak response amplitudes significantly decreased over the course of repeated exposure for both odors (Fig. 2*F*). We note that peak response amplitudes were not significantly different between Odor A- and Odor B-evoked responses across the 4 d, indicating no bias between the two odor mixtures (Fig. 2*G*). These findings indicate that repeated passive odor exposure reduces both the number of responsive PGCs and the amplitude of the odor-evoked responses while preserving the relative responsive properties to the odor mixtures. Thus, passive sensory experience leads to sparser and weaker odor representations. For reference, detailed statistical analyses for Figure 2 and Extended Data Figure 2-1 are provided in Extended Data Figure 2-2.

### Odor-cued Go/No-go discrimination learning

Having characterized PGC responses during passive odor exposure, we next investigated the dynamics of PGC responses during active odor experience. Mice were trained to perform a Go/No-go odor discrimination task while simultaneously recording PGC activity (Fig. 3*A*). After the recovery from craniotomy, mice underwent a pretraining period in which they were habituated to head fixation and water restriction and were trained to lick a port for water delivery. Following this period, they were trained on the odor-cued Go/No-go discrimination task.

10.1523/ENEURO.0171-26.2026.f3-1 Figure 3-1 **Statistical summary for Figure 3.** Summary of the sample size, statistical test, and exact *p*-values for the analyses shown in Figure 3. Download Figure 3-1, DOCX file.

In the discrimination task, each trial consisted of a 3 s odor presentation followed by a 2 s response period in which mice could lick the port (Fig. 3*B*). During Go odor trials, licking the port resulted in water delivery, whereas during No-go odor trials, licking led to punishment with aversive white noise (Fig. 3*C*).

We conducted the odor discrimination task in two phases: an easy discrimination phase using a distinct odor mixture pair (Go, 80% ET and 20% HP; No-go, 20% ET and 80% HP) and a difficult discrimination phase using a similar odor mixture pair (Go, 52% ET and 48% HP; No-go, 48% ET and 52% HP; Fig. 3*D*). This experimental design was used to examine neural activity during learning of the task rules in the easy discrimination task and during discrimination between highly similar odor cues with reward or punishment in the difficult discrimination task. In both phases, mice progressively improved their performance to reach an expert level within 4 d (Fig. 3*E*,*F*). For reference, detailed statistical analyses for Figure 3 are provided in Extended Data Figure 3-1.

### Active odor discrimination drives progressive enhancement of PGC responses

To characterize how PGC activity is modulated during active odor-guided behavior, we performed chronic two-photon calcium imaging to monitor PGC activity during the Go/No-go easy discrimination task (Fig. 4*A*). A subset of odor-responsive PGCs exhibited calcium transients during the 3 s odor period, which were mostly excitatory responses, and displayed distinct response patterns to Go and No-go odors. When the activity of all recorded PGCs was aligned, odor-specific and heterogeneous response patterns emerged within the responsive subpopulation (Fig. 4*B*). Both excitatory and inhibitory responses to odor stimuli were observed among individual PGCs (Fig. 4*C*). Furthermore, odor-responsive PGCs were distributed broadly across the imaging field and did not form spatial clusters, and this distribution remained stable over the course of training (Fig. 4*D*). There was no spatial clustering of Go odor- and No-go odor-responsive PGCs in the imaging field (Extended Data Fig. 4-1).

10.1523/ENEURO.0171-26.2026.f4-1 Figure 4-1 **Spatial organization of excitatory- and inhibitory-responsive PGCs during easy discrimination task.** Cumulative distributions of pairwise distances among excitatory (red) and inhibitory (blue) cells across Days 1–4 during easy discrimination task. Statistical comparisons between excitatory and inhibitory populations were performed using the Wilcoxon signed-rank test. Day 1, Go odor, *p* *=* 0.500, excitatory *n* = 326 cells, inhibitory *n* = 147 cells; No-go odor, *p* *=* 0.500, excitatory *n* = 278 cells, inhibitory *n* = 112 cells; Day 2, Go odor, *p* *=* 0.685, excitatory *n* = 266 cells, inhibitory *n* = 130 cells; No-go odor, *p* *=* 0.079, excitatory *n* = 219 cells, inhibitory *n* = 163 cells; Day 3, Go odor, *p* *=* 0.500, excitatory *n* = 282 cells, inhibitory n = 158 cells; No-go odor, *p* *=* 0.715, excitatory *n* = 268 cells, inhibitory n = 119 cells; Day 4, Go odor, *p* *=* 0.138, excitatory n = 301 cells, inhibitory *n* = 129 cells; No-go odor, *p* *=* 0.892, excitatory *n* = 216 cells, inhibitory *n* = 128 cells, from 5 mice. Detailed statistical analyses are provided in Figure 4-2. Download Figure 4-1, TIF file.

10.1523/ENEURO.0171-26.2026.f4-2 Figure 4-2 **Statistical summary for Figures 4 and 4-1.** Summary of the sample size, statistical test, degrees of freedom, exact *p*-values, and multiple comparisons for the analyses shown in Figures 4 and 4-1. Download Figure 4-2, DOCX file.

To quantify their response characteristics, we classified individual neurons based on their response profiles (excitatory or inhibitory) to Go and No-go odors. We found that the fraction of PGCs showing excitatory responses selective to Go odor significantly increased on Day 4 compared with Day 1 in a planned comparison, although the overall effect of training day was not significant by repeated-measures ANOVA, while the proportion of inhibitory-responsive cells remained relatively stable as ∼10–15% throughout training (Fig. 4*E*). We also tracked the response profiles of individual PGCs to visualize phenotype transitions across training (Fig. 4*F*). Tracking individual response patterns across training suggested that some nonresponsive cells acquired excitatory responses to Go odor. When we examined the peak response amplitudes of individual PGCs across learning, Go odor-evoked responses were significantly larger than No-go odor-evoked responses throughout learning (Fig. 4*G*). The weaker responses to the No-go odor likely reflect habituation to a nonrewarded stimulus, similar to the passive exposure condition, whereas Go odor responses are maintained or enhanced by the context and reward association. These findings indicate that active odor discrimination learning increased the proportion of odor-responsive PGCs, with a selective increase in excitatory responses to Go odor. In contrast to repeated passive odor exposure, active discrimination learning reorganized PGC activity toward an enhanced representation of the reward-associated odor. For reference, detailed statistical analyses for Figure 4 and Extended Data Figure 4-1 are provided in Extended Data Figure 4-2.

### Discriminating similar odors recruits and reorganizes PGC responses during learning

Next, to examine how PGC responses change when mice were trained to discriminate between highly similar odor pairs, we monitored PGC activity during the difficult discrimination task (Fig. 5*A*). This task was performed in the same mice, and PGC activity was recorded from the same field of view as in the easy discrimination task (Fig. 4). Compared with the easy discrimination task, the highly similar odor mixtures elicited weaker and less distinct responses (Fig. 5*B*). As in the easy discrimination task, both excitatory and inhibitory responses to odor stimuli were observed among individual PGCs (Fig. 5*C*). Odor-responsive PGCs were broadly distributed, and the number of cells responsive to either odor increased over the training period (Fig. 5*D*). There was no spatial clustering of Go odor- and No-go odor-responsive PGCs in the imaging field (Extended Data Fig. 5-1).

10.1523/ENEURO.0171-26.2026.f5-1 Figure 5-1 **Spatial organization of excitatory- and inhibitory-responsive PGCs during difficult discrimination task.** Cumulative distributions of pairwise distances among excitatory (red) and inhibitory (blue) cells across Days 1–4 during difficult discrimination task. Statistical comparisons between excitatory and inhibitory populations were performed using the Wilcoxon signed-rank test. Day 1, Go odor, *p* *=* 0.465, excitatory *n* = 148 cells, inhibitory *n* = 272 cells; No-go odor, *p* *=* 0.715, excitatory *n* = 194 cells, inhibitory *n* = 280 cells; Day 2, Go odor, *p* *=* 0.685, excitatory *n* = 275 cells, inhibitory *n* = 235 cells; No-go odor, *p* *=* 0.715, excitatory *n* = 250 cells, inhibitory *n* = 273 cells; Day 3, Go odor, *p* *=* 0.079, excitatory *n* = 268 cells, inhibitory *n* = 241 cells; No-go odor, *p* *=* 0.892, excitatory *n* = 242 cells, inhibitory *n* = 269 cells; Day 4, Go odor, *p* *=* 0.892, excitatory *n* = 263 cells, inhibitory *n* = 243 cells; No-go odor, *p* *=* 0.067, excitatory *n* = 236 cells, inhibitory *n* = 239 cells, from 5 mice. Detailed statistical analyses are provided in Figure 5-2. Download Figure 5-1, TIF file.

10.1523/ENEURO.0171-26.2026.f5-2 Figure 5-2 **Statistical summary for Figures 5 and 5-1.** Summary of the sample size, statistical test, degrees of freedom, exact *p*-values, and multiple comparisons for the analyses shown in Figures 5 and 5-1. Download Figure 5-2, DOCX file.

To quantify their response characteristics, we classified individual neurons based on their response profiles to Go and No-go odors, as in Figure 4 (Fig. 5*E*). The proportion of PGCs showing excitatory responses selective to Go odor tended to increase across training days. Tracking phenotype transitions of individual neurons suggested that some nonresponsive cells acquired excitatory responses to Go odor during training (Fig. 5*F*). When we examined the peak response amplitudes of individual PGCs, Go odor-evoked responses were larger than No-go odor-evoked responses across training days (Fig. 5*G*). These findings indicate that the difficult discrimination task recruits a broader population of PGCs and reorganizes their responses toward a more selective representation of the reward-associated odor. For reference, detailed statistical analyses for Figure 5 and Extended Data Figure 5-1 are provided in Extended Data Figure 5-2.

### Experience-dependent reorganization of PGC population coding

To examine how sensory experience under different task contexts reorganizes PGC population activity, we performed PCA and decoding analyses across the three experimental conditions: repeated passive odor exposure, the easy discrimination task, and the difficult discrimination task. During repeated passive odor exposure, the population trajectories evoked by Odor A and Odor B were clearly separated on Day 1 but showed less divergence on Day 4 (Fig. 6*A*). To quantify the separation between odor representations, we calculated the Euclidean distance between Odor A and Odor B trajectories in principal component space (Fig. 6*B*). Although the distance increased following odor onset and persisted during the odor period on all days, the separation on Day 4 increased more slowly and reached a lower peak than on Day 1. To further assess discriminability between Odor A and Odor B, we classified them using Linear SVC with different numbers of cells used for training. On both Day 1 and Day 4, decoding accuracy gradually improved as the number of cells increased and was significantly higher during the odor period than during the preodor period, indicating that odor information was maintained despite habituation (Fig. 6*G*,*H*). However, a larger number of neurons were required to reach ∼80% accuracy on Day 4 compared with Day 1 (Fig. 6*I*). These findings indicate that repeated passive odor exposure led to sparser and weaker odor representations while maintaining ensemble patterns for discrimination.

10.1523/ENEURO.0171-26.2026.f6-1 Figure 6-1 **Contribution of excitatory- and inhibitory-responsive PGCs to decoding performance across odor discrimination tasks**. **A,B**, Contributions of individual excitatory-responsive (red) and inhibitory-responsive (blue) cells during the easy discrimination task (A) and difficult discrimination task (B). Contribution was defined as the absolute value of the linear classifier weight of each neuron across cross-validation folds. Statistical comparisons were performed using Mann-Whitney U test. The numbers of analyzed cells and corresponding p values for each day are as follows: (A) Day 1, Go odor, *p* = 0.034, excitatory *n* = 438 cells, inhibitory *n* = 174 cells; No-go odor, *p* = 0.542, excitatory *n* = 357 cells, inhibitory *n* = 137 cells; Day 2, Go odor, *p* = 0.029, excitatory *n* = 355 cells, inhibitory *n* = 153 cells; No-go odor, *p* = 0.034, excitatory *n* = 296 cells, inhibitory *n* = 202 cells; Day 3, Go odor, *p* = 0.038, excitatory *n* = 358 cells, inhibitory *n* = 191 cells; No-go odor, *p* < 0.001, excitatory *n* = 335 cells, inhibitory *n* = 154 cells; Day 4, Go odor, *p* = 0.035, excitatory *n* = 374 cells, inhibitory *n* = 138 cells; No-go odor, *p* = 0.922, excitatory *n* = 270 cells, inhibitory *n* = 142 cells, from 5 mice. (B) Day 1, Go odor, *p* = 0.043, excitatory *n* = 206 cells, inhibitory *n* = 345 cells; No-go odor, *p* < 0.001, excitatory *n* = 278 cells, inhibitory *n* = 357 cells; Day 2, Go odor, *p* = 0.021, excitatory *n* = 372 cells, inhibitory *n* = 309 cells; No-go odor, *p* < 0.001, excitatory *n* = 355 cells, inhibitory *n* = 355 cells; Day 3, Go odor, *p* < 0.001, excitatory *n* = 321 cells, inhibitory *n* = 348 cells; No-go odor, *p* < 0.001, excitatory *n* = 321 cells, inhibitory *n* = 348 cells; Day 4, Go odor, *p* < 0.001, excitatory *n* = 390 cells, inhibitory *n* = 329 cells; No-go odor, *p* < 0.001, excitatory *n* = 357 cells, inhibitory *n* = 320 cells, from 5 mice. n.s., not significant; *p < 0.05; **p < 0.01; ***p < 0.001. Detailed statistical analyses are provided in Figure 6-2. Download Figure 6-1, TIF file.

10.1523/ENEURO.0171-26.2026.f6-2 Figure 6-2 **Statistical summary for Figures 6 and 6-1.** Summary of the sample size, statistical test, degrees of freedom, exact *p*-values, and multiple comparisons for the analyses shown in Figures 6 and 6-1. Download Figure 6-2, DOCX file.

In contrast, during the easy discrimination task, trajectory separation during the odor period was larger on Day 4 compared with Day 1 (Fig. 6*C*,*D*). Notably, the Euclidean distance also increased during the response period, likely reflecting motor- and reward-related signals. Despite the increase in the separation, decoding accuracy was already high on Day 1 and remained largely unchanged after learning, and the number of neurons required to reach ∼80% decoding accuracy was comparable between Day 1 and Day 4 (Fig. 6*J*–*L*). We also found that excitatory-responsive neurons tend to contribute more than inhibitory-responsive neurons, especially for the Go odor (Extended Data Fig. 6-1*A*). These findings indicate that active odor discrimination learning expanded the separation of Go/No-go population trajectories without increasing the required population size. This suggests that the newly recruited Go-responsive neurons are redundant with the existing population.

During the difficult discrimination task, PCA demonstrated that population trajectories evoked by the Go and No-go odors were more divergent on Day 4 compared with Day 1 (Fig. 6*E*). Consistent with this result, Euclidean distance between Go and No-go population trajectories was larger on Day 4 than on Day 1 (Fig. 6*F*). The decoding accuracy improved after learning, and the number of cells required to reach ∼80% decoding accuracy was significantly reduced on Day 4 compared with Day 1 (Fig. 6*M*–*O*). Comparison of decoder weights showed that excitatory-responsive neurons contributed more than inhibitory-responsive neurons for both Go and No-go odors throughout training (Extended Data Fig. 6-1*B*). Thus, under more demanding discrimination conditions, PGCs encode odor information more efficiently, achieving higher selectivity with smaller ensembles.

Taken together, these results indicate that PGC population coding is bidirectionally modulated by experience. Passive odor exposure leads to sparser and weaker odor representations while preserving decoding performance, whereas active learning selectively enhances and reorganizes PGC ensemble activity to support task-dependent odor discrimination. For reference, detailed statistical analyses for Figure 6 and Extended Data Figure 6-1 are provided in Extended Data Figure 6-2.

## Discussion

Here, we investigated the functional dynamics of PGCs in the OB using in vivo two-photon calcium imaging in awake mice. During passive odor exposure, although only a small subset of PGCs responded to odors, this sparse population activity was sufficient to decode odor identity. Repeated passive odor exposure reduced response amplitude and the number of odor-responsive PGCs while maintaining odor selectivity. In contrast, active odor discrimination increased the number of odor-responsive PGCs and enhanced the responses to reward-associated odors. To the best of our knowledge, this is the first demonstration that experience-dependent learning bidirectionally modulates PGC activity in the OB. These results reveal the functional heterogeneity and plasticity in the glomerular inhibitory microcircuits and show that PGCs dynamically modulate their activity to facilitate adaptive odor processing within the OB circuit.

Odor representations in the OB are spatially organized patterns based on OSN inputs, with individual odorants activating a specific subset of glomeruli (Uchida et al., 2000). In our study, passive odor exposure induced a small but consistent odor-responsive population of PGCs, and ensemble activity contained sufficient information to discriminate among odorants. Importantly, individual PGCs responded to chemically distinct odorants without apparent structural selectivity. Because imaging was restricted to the dorsal part of the posterior OB, region-specific selectivity may not be captured. Another possibility is that the sparse PGC responses reflect a feature of population coding, in which odor identity emerges from neuronal ensembles rather than individual cell selectivity. This interpretation is consistent with previous studies showing sparse odor representations of M/T cells in the OB, suggesting that sparse and distributed ensemble activity can efficiently represent diverse odor identities (Rinberg et al., 2006a; Davison and Katz, 2007; Yu et al., 2013; Gschwend et al., 2016). This sparse coding strategy may enhance the capacity to represent a wide variety of odors.

We observed distinct differences in PGC responses between passive and active odor experiences. Repeated passive odor exposure led to a reduction in the proportion of odor-responsive PGCs and their response amplitude while odor selectivity was preserved. This phenomenon resembles sensory habituation observed in other sensory systems and contributes to the stabilization of odor representations during repetitive sensory experience (Kato et al., 2012). Because PGCs provide inhibitory input to M/T cells, a reduction in PGC activity alone would be expected to disinhibit, rather than reduce, M/T cell responses. Our data are therefore inconsistent with a simple model in which reduced PGC inhibition accounts for the M/T cell habituation reported previously (Kato et al., 2012). One possible explanation is that repeated passive odor exposure induces adaptation in OSNs (Ogg et al., 2015; Kass et al., 2016), although another study showed that OSN inputs remained stable during repeated passive odor exposure (Kato et al., 2012). Such peripheral adaptation would reduce excitatory input to the glomerular circuit, leading to decreased activity in both PGCs and M/T cells. Thus, the concurrent reduction in PGC and M/T cell activity during passive odor exposure may partly reflect reduced upstream sensory input, although the contribution of upstream adaptation remains to be fully resolved. During active odor discrimination, however, the fraction of odor-responsive PGCs was maintained or increased, and responses to Go odor were selectively enhanced. This learning-dependent PGC plasticity could reshape the inhibition of M/T cells, refining their odor tuning and enhancing the signal-to-noise ratio for reward-related odor mixtures. The weak response to No-go odor reflects the habituation process observed during passive repeated exposure because the No-go odor is not associated with reward. In contrast, the Go response is selectively maintained or enhanced because of its predictive value for a reward. In the easy discrimination task, we observed that the Euclidean distance between Go and No-go trajectories in PCA space increased with training, whereas decoding accuracy changed little. These metrics capture different aspects of population activity. The PCA distance is sensitive to the magnitude of responses. Therefore, an increase in neurons showing excitatory responses selectively to Go odor can expand the distance between trajectories without changing separability. In contrast, linear decoding reflects separability. Because Go and No-go responses were already well separated at the beginning of training, decoding accuracy was already high on Day 1 and remained largely unchanged after learning.

When the odor mixtures were highly similar, the population of responsive cells expanded. Despite the recruitment of additional PGCs, the difficult discrimination task showed slower and smaller trajectory separation between Go and No-go odors compared with the easy discrimination task. The peak of the Euclidean distance between population trajectories for Go odor and No-go odor emerged at the end of the response period rather than during the odor period. It was reported that olfactory decisions can be made within a few hundred milliseconds after odor onset (Uchida and Mainen, 2003; Abraham et al., 2004; Rinberg et al., 2006b; Wesson et al., 2008). Therefore, activity during the response period is unlikely to reflect sensory decision alone and may include licking-related motor signals and reward-related signals.

It has been reported that PGCs receive top–down inputs from the olfactory cortex, including the anterior olfactory nucleus and the piriform cortex (Boyd et al., 2012; Wen et al., 2019). In addition, PGCs also receive inhibitory inputs directly from the basal forebrain and olfactory cortex (Sanz Diez et al., 2019; De Saint Jan, 2022; Mazo et al., 2022). These inhibitory inputs may be preferentially recruited when discriminating highly similar odor mixtures, potentially contributing to the prominent inhibitory responses observed during the early phase of the difficult discrimination task. These centrifugal inputs convey contextual information, such as reward or punishment cues, and may bias PGC activity toward reward-associated odors. Such task-dependent modulation reflects the integration of bottom–up sensory inputs from OSNs, top–down cortical feedback including contextual information, and neuromodulators such as dopaminergic and cholinergic signals related to reward (Kato et al., 2023; Yu et al., 2025). Furthermore, a subset of PGCs corelease GABA and dopamine (Ennis et al., 2001; Liu et al., 2013; Galliano et al., 2018; Kosaka et al., 2020; Capsoni et al., 2021; Lyons-Warren et al., 2023). Dopamine release from PGCs enhances the contrast between glomerular odor representations by modulating synaptic strength between OSNs and M/T cells (Ennis et al., 2001; Liu et al., 2013). Most neurons in the OB express either or both D1-type and D2-type dopamine receptors (Yu et al., 2019), and pharmacological manipulation of dopaminergic signaling alters odor detection thresholds and discrimination performance (Yue et al., 2004; Wei et al., 2006; Escanilla et al., 2009; Lyons-Warren et al., 2023). Thus, task-dependent modulation of PGC activity may involve both GABAergic inhibition and dopaminergic neuromodulation.

Previous studies also showed that OSNs exhibit stable responses even during an active discrimination task (Chu et al., 2017), whereas M/T cells undergo task-dependent pattern separation (Chu et al., 2016; Yamada et al., 2017). Because OSN adaptation alone would be expected to reduce rather than enhance odor-evoked activity, the selective enhancement of Go odor responses during active discrimination likely involves additional mechanisms, including top–down modulation and local synaptic plasticity. In addition, several studies showed that experience and learning can reorganize odor representations in M/T cells to become sparser and more selective (Kato et al., 2012; Vinograd et al., 2017; Kudryavitskaya et al., 2021). Our findings suggest that PGCs may contribute to the sparsification and refinement of M/T cell representations by integrating sensory inputs, neuromodulatory signals, and centrifugal cortical feedback. Specifically, PGCs serve as an intermediate processing gate that transforms stable sensory inputs into flexible, experience-dependent representations in M/T cells. Through the integration of bottom–up sensory inputs and top–down cortical inputs, PGCs can dynamically modulate their activity and selectivity as well as the temporal dynamics of M/T cell responses.

We did not distinguish between PGC subtypes based on their structural, physiological, or genetic properties (Kosaka and Kosaka, 2005, 2007; Parrish-Aungst et al., 2007; Kiyokage et al., 2010; Nagayama et al., 2014; Capsoni et al., 2021). Different PGC subtypes may play distinct roles in shaping odor representations, and their dynamics could differ significantly. In addition, our study did not demonstrate causal relationships between PGC activity changes and behavioral performance. Optogenetic or chemogenetic manipulations of specific PGC populations during learning would be required to determine whether such plasticity directly contributes to odor discrimination performance. Moreover, although our chronic imaging revealed experience-dependent plasticity in PGC population activity across days, it did not identify the synaptic or circuit mechanisms underlying the emergence of these response patterns. Electrophysiological recordings and circuit perturbations will be important in future studies to elucidate the mechanisms. We also note that we used the mDlx enhancer to drive GCaMP6f broadly in GABAergic neurons (Dimidschstein et al., 2016). Therefore, our imaging may include not only PGCs but also other GABAergic interneurons, such as short-axon cells (SACs). However, these SACs are comparatively sparse (Parrish-Aungst et al., 2007; Kiyokage et al., 2010). Thus, the majority of our recorded neurons are likely to be PGCs with a possible contribution from a small number of SACs. Further investigations will be necessary for a comprehensive understanding of how PGCs support experience-dependent sensory processing in the OB circuit.

## Synthesis

Reviewing Editor: Arianna Maffei, Stony Brook University Decisions are customarily a result of the Reviewing Editor and the peer reviewers coming together and discussing their recommendations until a consensus is reached. When revisions are invited, a fact-based synthesis statement explaining their decision and outlining what is needed to prepare a revision will be listed below. The following reviewer(s) agreed to reveal their identity: NONE. Note: If this manuscript was transferred from JNeurosci and a decision was made to accept the manuscript without peer review, a synthesis may not be available.

The authors have thoroughly addressed the reviewers' feedback and improved the manuscript. I do not have additional concerns.
