# Supplementary figures and images for: Experience-Dependent Plasticity of Periglomerular Cells in the Olfactory Bulb
Source: eNeuro. 2026 Jul 14;13(7):ENEURO.0171-26.2026. doi: 10.1523/ENEURO.0171-26.2026 (PMC13379368; doi:10.1523/ENEURO.0171-26.2026)

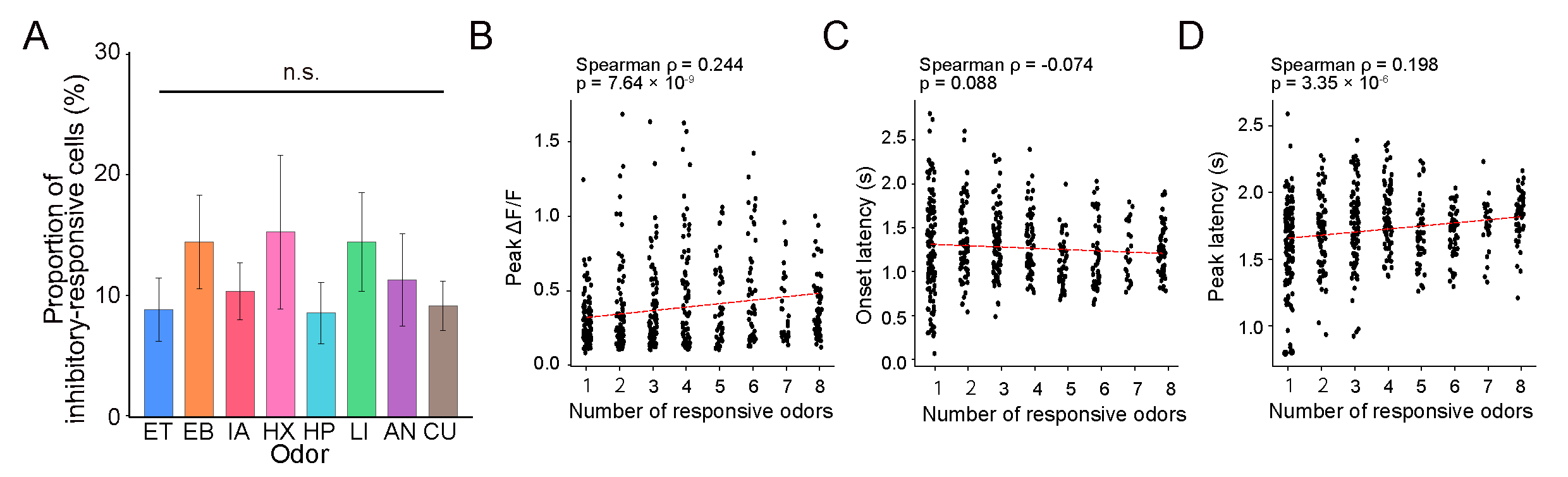

Supplement: Figure 1-1 — Population activity dynamics and variability. A, Proportion of PGCs showing inhibitory responses to each odorant. n = 821 cells from 4 mice. Repeated-measures ANOVA, F (7, 21) = 0.599, p = 0.749. B–D, Relationship between the number of responsive odors per neuron and peak response amplitude (B), response onset latency (C), and peak latency (D). Each dot represents an individual neuron. Spearman’s rank correlation coefficients (ρ) and corresponding p-values are indicated in each panel. Error bars denote the s.e.m. n.s., not significant. Detailed statistical analyses are provided in Figure 1-2. Download Figure 1-1, TIF file. [file eneuro-13-ENEURO.0171-26.2026-s002.tif]

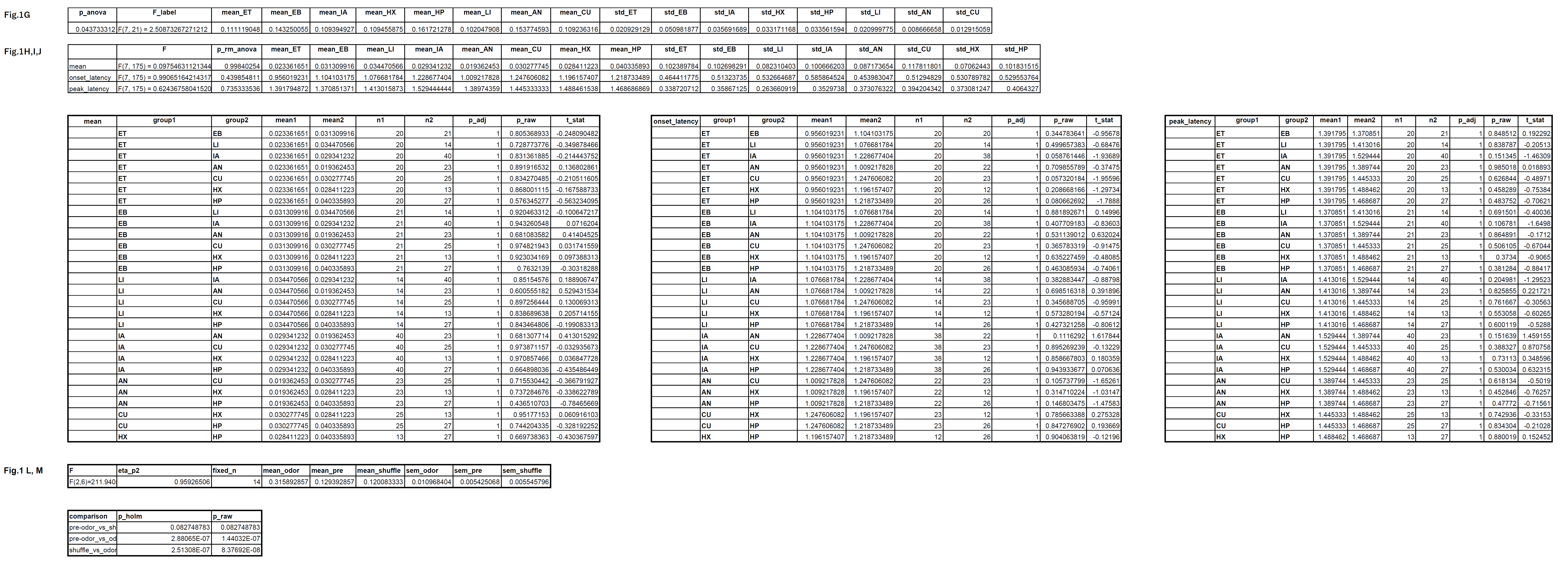

Supplement: Figure 1-2 — Statistical summary for Figures 1 and 1-1. Summary of the sample size, statistical test, degrees of freedom, exact p-values, and multiple comparisons for the analyses shown in Figures 1 and 1-1. Download Figure 1-2, DOCX file. [file eneuro-13-ENEURO.0171-26.2026-s003.docx]

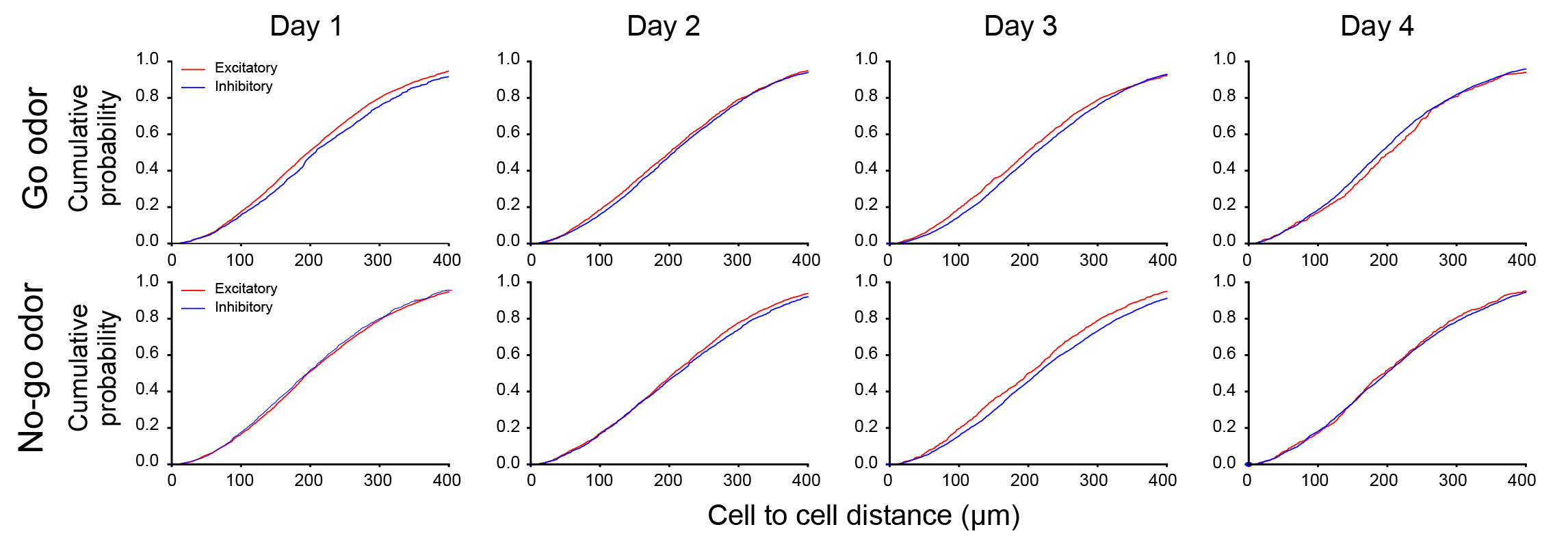

Supplement: Figure 2-1 — Spatial organization of excitatory- and inhibitory-responsive PGCs during repeated passive odor exposure. Cumulative distributions of pairwise distances among excitatory (red) and inhibitory (blue) cells across Days 1–4 during repeated passive odor exposure. Statistical comparisons between excitatory and inhibitory populations were performed using the Wilcoxon signed-rank test. Day 1, Odor A, p = 0.138, excitatory n = 543 cells, inhibitory n = 303 cells; Odor B, p = 0.500, excitatory n = 478 cells, inhibitory n = 303 cells; Day 2, Odor A, p = 0.685, excitatory n = 303 cells, inhibitory n = 555 cells; Odor B, p = 0.892, excitatory n = 304 cells, inhibitory n = 458 cells; Day 3, Odor A, p = 0.079, excitatory n = 238 cells, inhibitory n = 544 cells; Odor B, p = 0.685, excitatory n = 260 cells, inhibitory n = 454 cells; Day 4, Odor A, p = 0.138, excitatory n = 162 cells, inhibitory n = 521 cells; Odor B, p = 0.892, excitatory n = 229 cells, inhibitory n = 422 cells, from 5 mice. Detailed statistical analyses are provided in Figure 2-2. Download Figure 2-1, TIF file. [file eneuro-13-ENEURO.0171-26.2026-s004.tif]

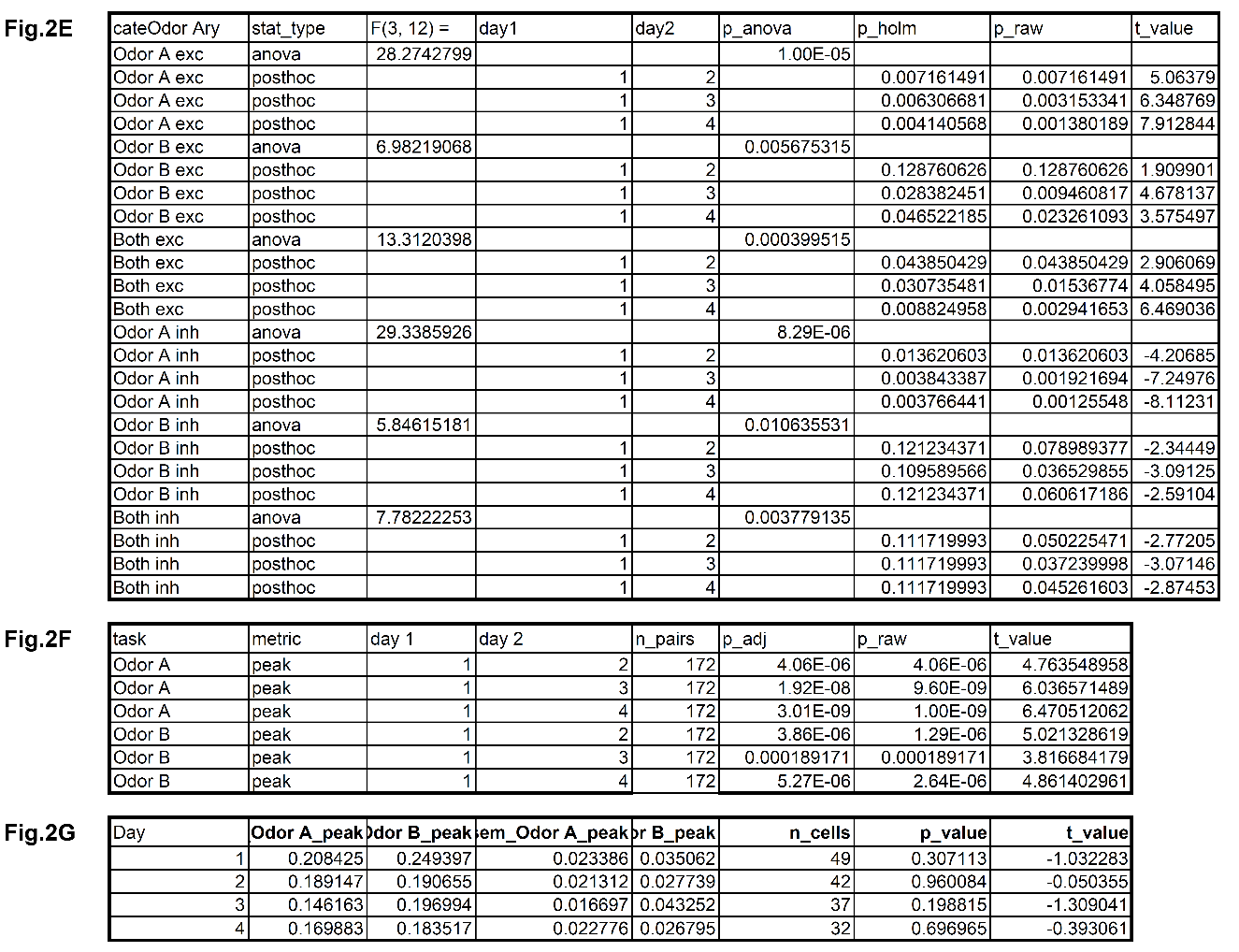

Supplement: Figure 2-2 — Statistical summary for Figures 2 and 2-1. Summary of the sample size, statistical test, degrees of freedom, exact p-values, and multiple comparisons for the analyses shown in Figures 2 and 2-1. Download Figure 2-2, DOCX file. [file eneuro-13-ENEURO.0171-26.2026-s005.docx]

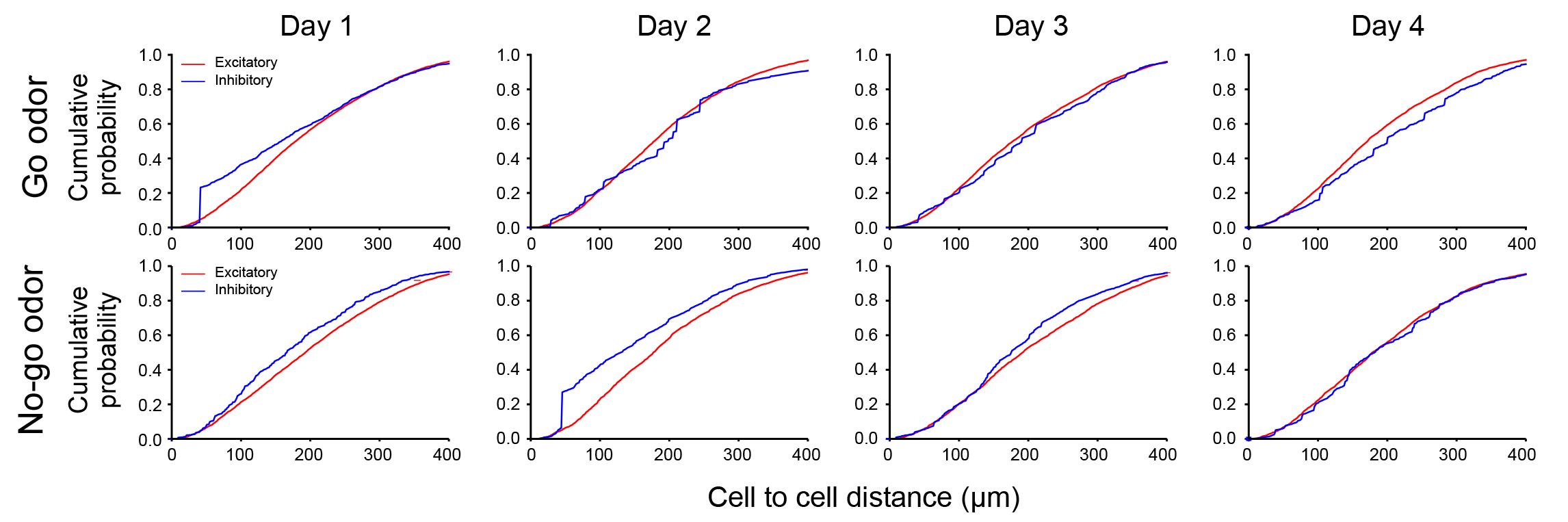

Supplement: Figure 4-1 — Spatial organization of excitatory- and inhibitory-responsive PGCs during easy discrimination task. Cumulative distributions of pairwise distances among excitatory (red) and inhibitory (blue) cells across Days 1–4 during easy discrimination task. Statistical comparisons between excitatory and inhibitory populations were performed using the Wilcoxon signed-rank test. Day 1, Go odor, p = 0.500, excitatory n = 326 cells, inhibitory n = 147 cells; No-go odor, p = 0.500, excitatory n = 278 cells, inhibitory n = 112 cells; Day 2, Go odor, p = 0.685, excitatory n = 266 cells, inhibitory n = 130 cells; No-go odor, p = 0.079, excitatory n = 219 cells, inhibitory n = 163 cells; Day 3, Go odor, p = 0.500, excitatory n = 282 cells, inhibitory n = 158 cells; No-go odor, p = 0.715, excitatory n = 268 cells, inhibitory n = 119 cells; Day 4, Go odor, p = 0.138, excitatory n = 301 cells, inhibitory n = 129 cells; No-go odor, p = 0.892, excitatory n = 216 cells, inhibitory n = 128 cells, from 5 mice. Detailed statistical analyses are provided in Figure 4-2. Download Figure 4-1, TIF file. [file eneuro-13-ENEURO.0171-26.2026-s007.tif]

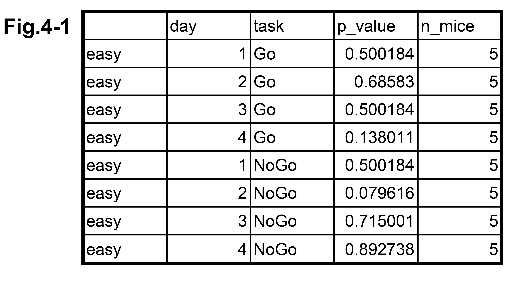

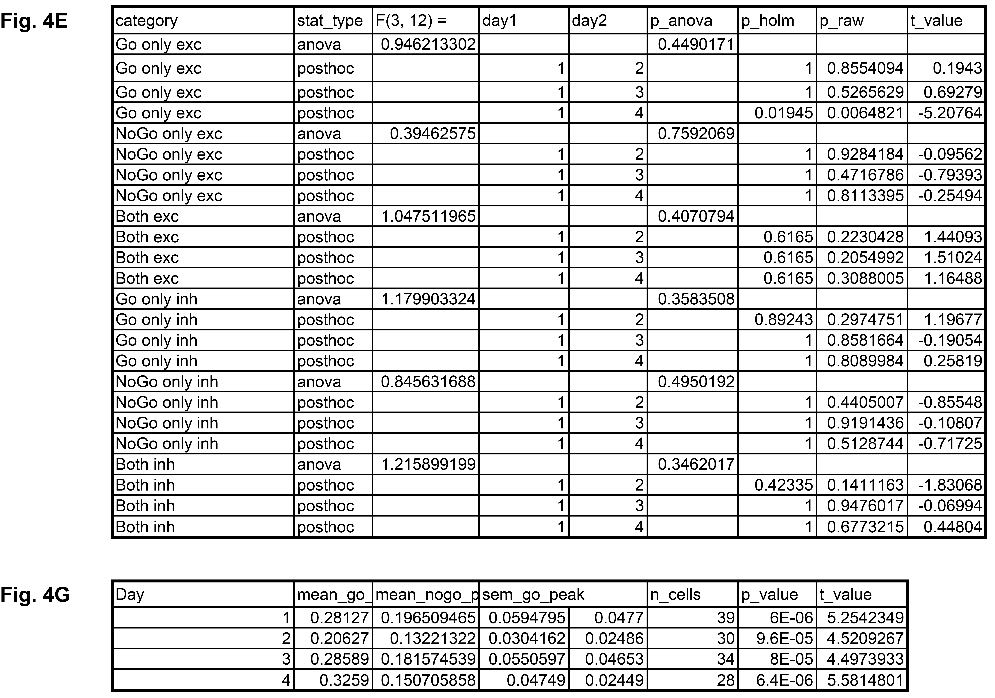

Supplement: Figure 4-2 — Statistical summary for Figures 4 and 4-1. Summary of the sample size, statistical test, degrees of freedom, exact p-values, and multiple comparisons for the analyses shown in Figures 4 and 4-1. Download Figure 4-2, DOCX file. [file eneuro-13-ENEURO.0171-26.2026-s008.docx]

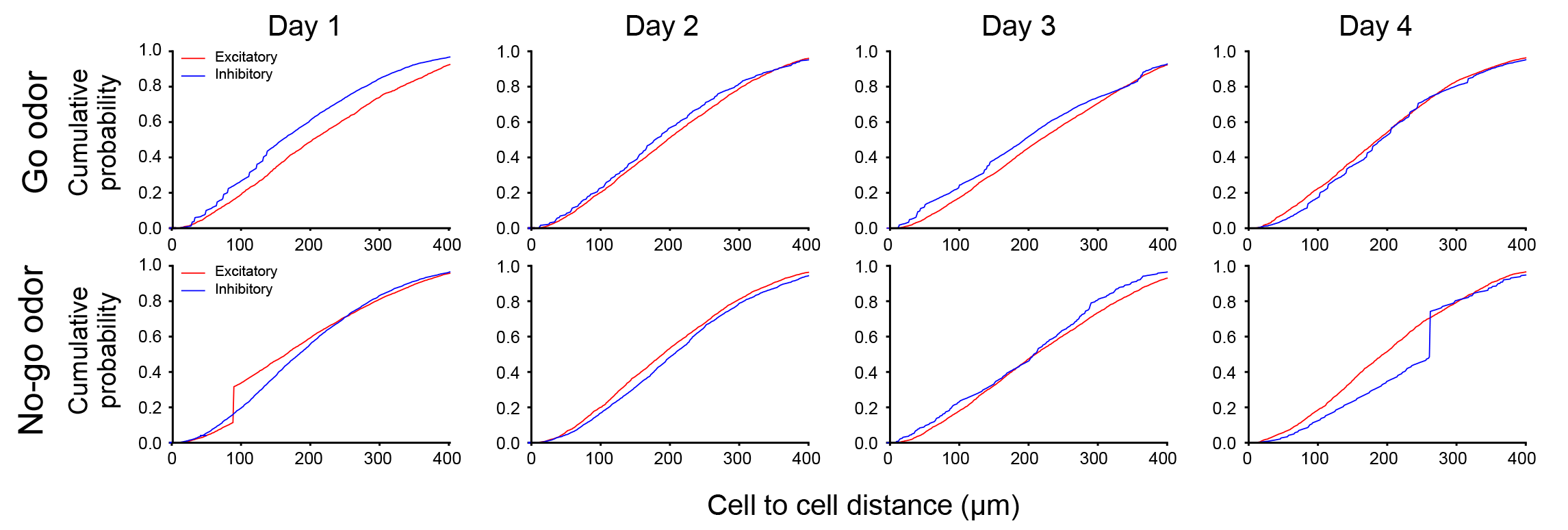

Supplement: Figure 5-1 — Spatial organization of excitatory- and inhibitory-responsive PGCs during difficult discrimination task. Cumulative distributions of pairwise distances among excitatory (red) and inhibitory (blue) cells across Days 1–4 during difficult discrimination task. Statistical comparisons between excitatory and inhibitory populations were performed using the Wilcoxon signed-rank test. Day 1, Go odor, p = 0.465, excitatory n = 148 cells, inhibitory n = 272 cells; No-go odor, p = 0.715, excitatory n = 194 cells, inhibitory n = 280 cells; Day 2, Go odor, p = 0.685, excitatory n = 275 cells, inhibitory n = 235 cells; No-go odor, p = 0.715, excitatory n = 250 cells, inhibitory n = 273 cells; Day 3, Go odor, p = 0.079, excitatory n = 268 cells, inhibitory n = 241 cells; No-go odor, p = 0.892, excitatory n = 242 cells, inhibitory n = 269 cells; Day 4, Go odor, p = 0.892, excitatory n = 263 cells, inhibitory n = 243 cells; No-go odor, p = 0.067, excitatory n = 236 cells, inhibitory n = 239 cells, from 5 mice. Detailed statistical analyses are provided in Figure 5-2. Download Figure 5-1, TIF file. [file eneuro-13-ENEURO.0171-26.2026-s009.tif]

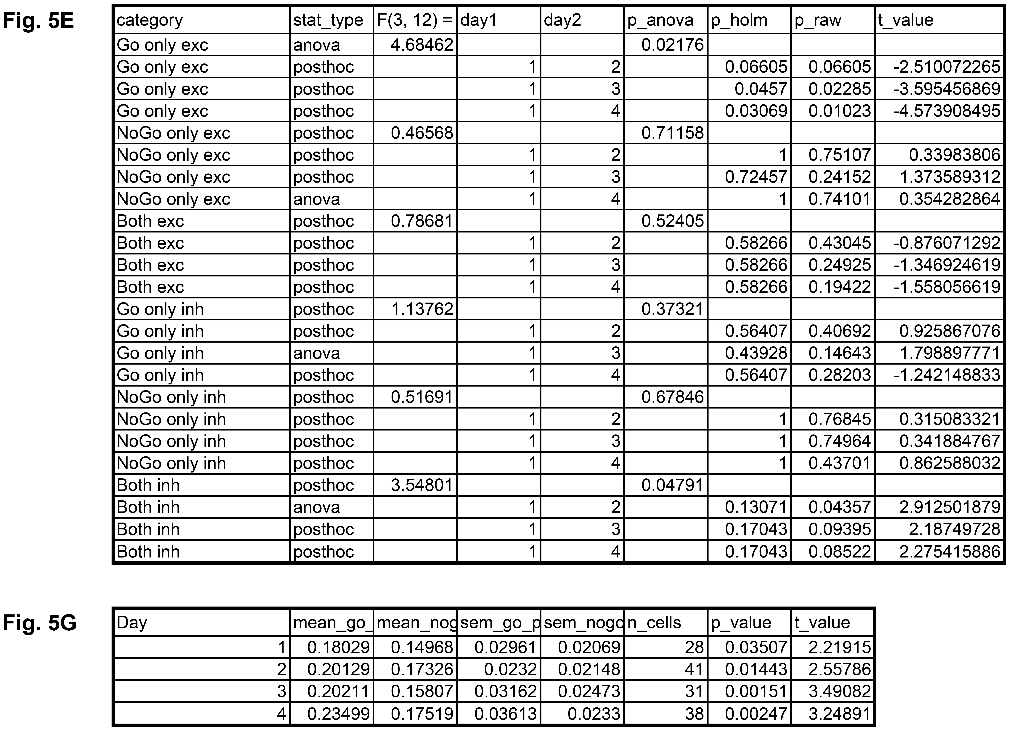

Supplement: Figure 5-2 — Statistical summary for Figures 5 and 5-1. Summary of the sample size, statistical test, degrees of freedom, exact p-values, and multiple comparisons for the analyses shown in Figures 5 and 5-1. Download Figure 5-2, DOCX file. [file eneuro-13-ENEURO.0171-26.2026-s010.docx]

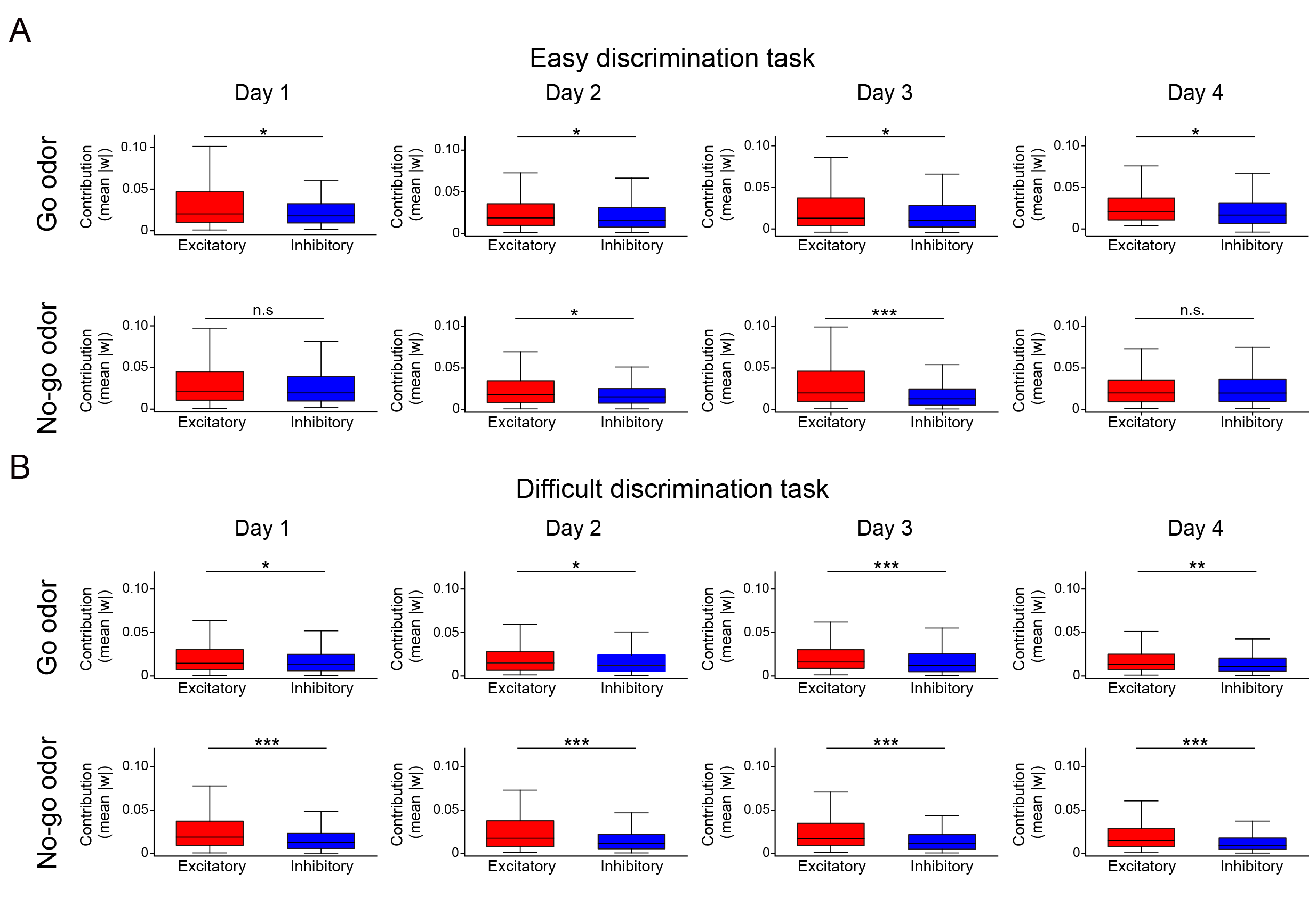

Supplement: Figure 6-1 — Contribution of excitatory- and inhibitory-responsive PGCs to decoding performance across odor discrimination tasks. A,B, Contributions of individual excitatory-responsive (red) and inhibitory-responsive (blue) cells during the easy discrimination task (A) and difficult discrimination task (B). Contribution was defined as the absolute value of the linear classifier weight of each neuron across cross-validation folds. Statistical comparisons were performed using Mann-Whitney U test. The numbers of analyzed cells and corresponding p values for each day are as follows: (A) Day 1, Go odor, p = 0.034, excitatory n = 438 cells, inhibitory n = 174 cells; No-go odor, p = 0.542, excitatory n = 357 cells, inhibitory n = 137 cells; Day 2, Go odor, p = 0.029, excitatory n = 355 cells, inhibitory n = 153 cells; No-go odor, p = 0.034, excitatory n = 296 cells, inhibitory n = 202 cells; Day 3, Go odor, p = 0.038, excitatory n = 358 cells, inhibitory n = 191 cells; No-go odor, p < 0.001, excitatory n = 335 cells, inhibitory n = 154 cells; Day 4, Go odor, p = 0.035, excitatory n = 374 cells, inhibitory n = 138 cells; No-go odor, p = 0.922, excitatory n = 270 cells, inhibitory n = 142 cells, from 5 mice. (B) Day 1, Go odor, p = 0.043, excitatory n = 206 cells, inhibitory n = 345 cells; No-go odor, p < 0.001, excitatory n = 278 cells, inhibitory n = 357 cells; Day 2, Go odor, p = 0.021, excitatory n = 372 cells, inhibitory n = 309 cells; No-go odor, p < 0.001, excitatory n = 355 cells, inhibitory n = 355 cells; Day 3, Go odor, p < 0.001, excitatory n = 321 cells, inhibitory n = 348 cells; No-go odor, p < 0.001, excitatory n = 321 cells, inhibitory n = 348 cells; Day 4, Go odor, p < 0.001, excitatory n = 390 cells, inhibitory n = 329 cells; No-go odor, p < 0.001, excitatory n = 357 cells, inhibitory n = 320 cells, from 5 mice. n.s., not significant; *p < 0.05; **p < 0.01; ***p < 0.001. Detailed statistical analyses are provided in Figure 6-2. Download Figure 6-1, TIF file. [file eneuro-13-ENEURO.0171-26.2026-s011.tif]

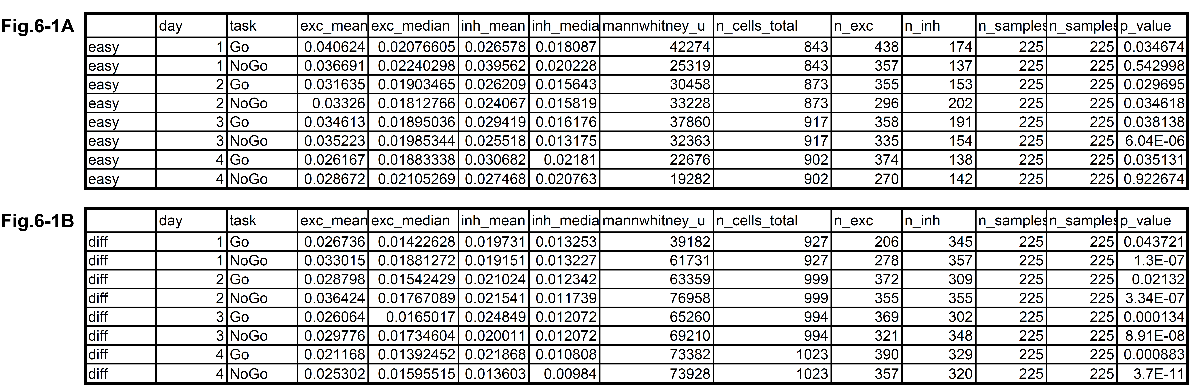

Supplement: Figure 6-2 — Statistical summary for Figures 6 and 6-1. Summary of the sample size, statistical test, degrees of freedom, exact p-values, and multiple comparisons for the analyses shown in Figures 6 and 6-1. Download Figure 6-2, DOCX file. [file eneuro-13-ENEURO.0171-26.2026-s012.docx]
