# Supplementary material for: Experience-Dependent Plasticity of Periglomerular Cells in the Olfactory Bulb
Source: eNeuro. 2026 Jul 14;13(7):ENEURO.0171-26.2026. doi: 10.1523/ENEURO.0171-26.2026 (PMC13379368; doi:10.1523/ENEURO.0171-26.2026)
Supplement: Supplementary file 2 [file eneuro-13-ENEURO.0171-26.2026-s006.docx]

Fig.3E

	mean	std		
First day	0.27969	0.195947		t (4) = -4.118, p = 0.014631393
Last day	0.850082	0.18005		

Fig.3F

	mean	std		
First day	0.193612	0.088285		t (4) = -6.893, p = 0.0023217325
Last day	0.764097	0.132662		
